# Osteoblast‐CD4^+^ CTL Crosstalk Mediated by SIRT1/DAAM2 Axis Prevents Age‐Related Bone Loss

**DOI:** 10.1002/advs.202501170

**Published:** 2025-07-26

**Authors:** Bin Yang, Guofu Zhang, Yizhou Zhu, Jingcheng Wang, Xinmin Feng, Wenyong Fei, Jihang Dai, Le Hu, Yi Zhang, Jun Cai, Binjia Ruan, Yue Jin, Fanhao Wei, Gang Lu, Dongan Wang, Jason Pui Yin Cheung, Graham Ka Hon Shea, Hao Chen, Kelvin Wai Kwok Yeung, Lei Wang, Yongxiang Wang

**Affiliations:** ^1^ Department of Orthopaedics Northern Jiangsu People's Hospital Affiliated to Yangzhou University Yangzhou 225001 China; ^2^ Northern Jiangsu People's Hospital Yangzhou 225001 China; ^3^ Northern Jiangsu People's Hospital, Clinical Teaching Hospital of Medical School Nanjing University Yangzhou 225001 China; ^4^ Department of Orthopaedics and Traumatology, School of Clinical Medicine, LKS Faculty of Medicine The University of Hong Kong Hong Kong SAR 999077 China; ^5^ Department of Medical Research Center Northern Jiangsu People's Hospital Affiliated to Yangzhou University Yangzhou 225001 China; ^6^ The Yangzhou School of Clinical Medicine of Dalian Medical University Yangzhou 225001 China; ^7^ CUHK‐SDU Joint Laboratory on Reproductive Genetics, School of Biomedical Sciences The Chinese University of Hong Kong Hong Kong 999077 China; ^8^ Department of Biomedical Engineering City University of Hong Kong Hong Kong SAR 999077 China; ^9^ Institute of Translational Medicine Medical College Yangzhou University Yangzhou 225000 China; ^10^ Yangzhou Key Laboratory of Regenerative Medicine Yangzhou 225001 China

**Keywords:** bone homeostasis, CD4^+^ cytotoxic T lymphocyte, chemokine signalling, DAAM2, osteoblast, osteoporosis, SIRT1

## Abstract

The dynamic production and clearance of senescent osteoblasts affects bone homeostasis and health. However, the relationship between senescent osteoblasts and the immune system remains unclear. Here, a landscape of the interaction between immune cells and osteoblasts through spatial analysis of the bone microenvironment is presented. Sirtuin 1 (SIRT1), a longevity gene, regulates bone mass maintenance through a mechanism involving osteoblast‐CD4^+^ cytotoxic T lymphocyte (CTL) crosstalk. In the osteoblastic niche, SIRT1 promotes the secretion of crucial chemokines, such as C‐C motif chemokine ligand 3 (CCL3), C‐C motif chemokine ligand 5 (CCL5), and C‐X‐C motif chemokine ligand 10 (CXCL10), by upregulating dishevelled‐associated activator of morphogenesis 2 (DAAM2) through the acetylation of enhancer of zeste homolog 2 (EZH2), activating and recruiting CD4^+^ CTLs that eliminate senescent osteoblasts in a major histocompatibility complex class II (MHC‐II)‐dependent manner, slowing the bone ageing process and ameliorating osteoporosis. DAAM2 serves as a pivotal downstream effector for SIRT1 to exert immune‐regulatory effects in the bone microenvironment; thus, targeting DAAM2 can treat osteoporosis by increasing CD4^+^ CTL responses. These results will facilitate the development of customised therapies targeting senescent osteoblasts to maintain bone health.

## Graphical abstract

In the osteoblastic niche, SIRT1 activates and recruits CD4^+^ CTLs by increasing DAAM2 expression via EZH2 deacetylation and boosting the secretion of key chemokines, such as CCL3, CCL5, and CXCL10. Then, CD4^+^ CTL directly eliminates senescent osteoblasts in an MHC‐II‐dependent way, thereby slowing down the process of bone ageing and effectively alleviating osteoporosis.

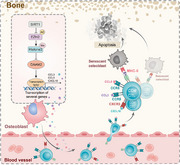

## 1 Introduction

Eliminating senescent cells is believed to prevent or delay age‐related bone loss and multiple comorbidities, such as cardiovascular dysfunction, cancer, osteoarthritis, and liver and lung fibrosis.^[^ ^1^, ^2^, ^3^, ^4^, ^5^, ^6^ ^]^ The osteoblastic dysfunction associated with ageing is the primary cause of age‐related bone loss.^[^ ^7^, ^8^ ^]^ The immune system, a key regulator of ageing, undergoes senescence and reduces its resistance to viruses and cancer cells, leading to related diseases.^[^ ^9^ ^]^ However, the relationship between senescent osteoblasts and the immune system remains unclear.

Recent studies have identified an unusual subpopulation of lymphocytes called CD4^+^ cytotoxic T lymphocytes (CD4^+^ CTLs) in the mammalian immune system that are crucial in combating viral infections and eliminating senescent or cancerous cells.^[^ ^10^, ^11^, ^12^ ^]^ An analysis of the immune system of supercentenarians revealed a significantly higher percentage of CD4^+^ CTLs in individuals over 110 years of age (≈25.3% of total T cells) than that of young controls (≈2.8%). In contrast, the overall T cell count was similar in both groups.^[^ ^13^ ^]^ A recent study revealed that a higher number of CD4^+^ CTLs is correlated with fewer senescent fibroblasts in aged skin.^[^ ^10^ ^]^ Hence, a robust anti‐ageing capacity is associated with enhanced immune surveillance against specific diseases.

The sirtuin family of proteins is essential in cellular resilience, energy metabolism, and aging, and has been denominated as the “longevity family of proteins”.^[^ ^14^ ^]^ All seven members of the sirtuin family (SIRT1‐7) have nicotinamide adenine dinucleotide (NAD^+^‐)‐binding and relatively conserved catalytic structural domains but differ in tissue distribution, subcellular localisation, enzymatic activity, and target proteins.^[^ ^15^ ^]^ Among them, SIRT1, SIRT6, and SIRT7 are expressed in bone tissue. Furthermore, SIRT1 was significantly down‐regulated in osteoporotic (OP) bone tissues.^[^ ^16^ ^]^ In addition, a study using a knockout mouse model indicated that bone mass significantly declined after SIRT7 knockdown.^[^ ^17^ ^]^ Recent investigations have found that the Sirtuin family of proteins is also closely linked with the regulation of immunity, especially that associated with T‐cells.^[^ ^18^ ^]^ SIRT1 directly or indirectly regulates immune responses by deacetylating key transcription factors.^[^ ^18^ ^]^ Our spatial analyses of human bone tissue samples at the outset indicated a close relationship between CD4^+^ CTLs and senescent osteoblasts. However, it requires further investigation on whether and how SIRT1 exerts a non‐cell‐autonomous function by regulating the osteoblast‐CD4^+^ CTL crosstalk.

In this work, we created osteoblast‐specific SIRT1‐knockout mice and conducted experiments involving the supplementation of CD4^+^ CTLs or the overexpression of DAAM2 in vivo. Our results demonstrate that DAAM2 is crucial as a downstream effector of SIRT1 in modulating immune responses within the bone microenvironment. Furthermore, targeting DAAM2 shows promise as a precise therapeutic approach for treating osteoporosis (OP) by enhancing CD4^+^ CTL responses.

## 2 Results

### 2.1 The Spatial Distribution of Immune Cell Subsets is Related to Senescent Osteoblasts

**Figure 1:**
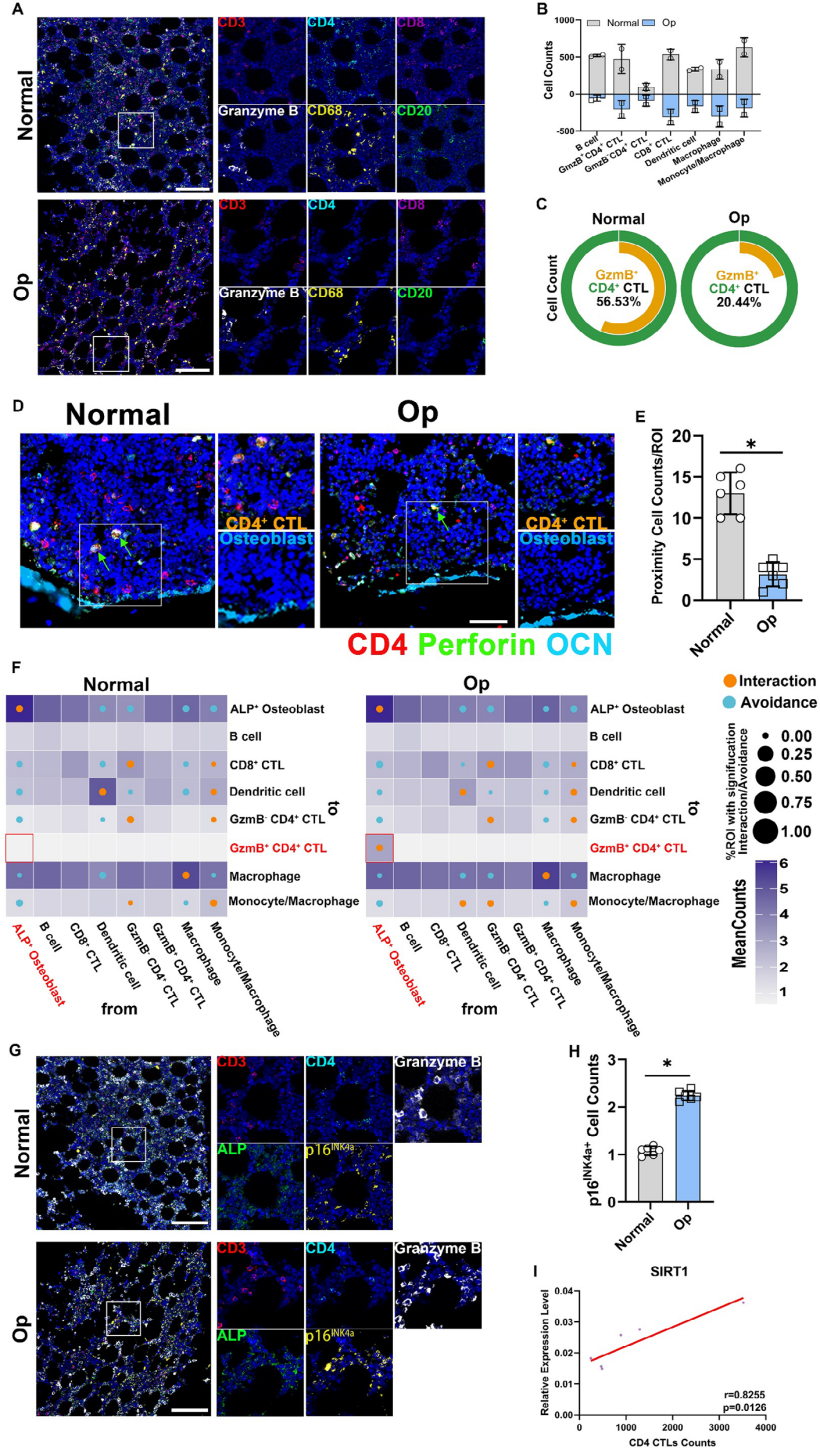
Distribution of immune cell subsets in the human bone microenvironment is closely linked to senescent osteoblasts by IMC analysis A) Merge and single colour staining of the indicated markers above each plot (except for blue staining of the nucleus) (scale bar, 200 µm). B) Counting of different immune cells in the ROI. C) Proportion of Granzyme B expression in CD4^+^ T cells. D,E) Representative IF staining and quantitative analyses of CD4 and Perforin in human normal and OP bone samples. CD4^+^ cells are CD3^+^ *T* cells (scale bar, 50 µm). F) Circles indicating patterns of cell–cell interactions/avoidance for normal and OP (Normal, *n* = 6; Op, *n* = 6). G,H) Merge and single colour staining of the indicated markers above each plot (except for blue staining of the nucleus) (scale bar, 200 µm). I) Correlation of SIRT1 with CD4^+^ CTLs. Data are compared with the control group as the mean ± SD. Statistical significance: ^*^ *p* < 0.05.

Immune cells play a pivotal role in regulating bone homeostasis. To ascertain whether the distribution of immune cell subsets in human bone tissue is altered with bone ageing, we conducted imaging mass cytometry (IMC) on bone tissue samples of various densities from older adult males aged > 60 years (Figure S1A, Supporting Information). We employed a supervised lineage assignment approach to classify osteoblasts and more than seven immune cell populations using canonical identity markers. Here, we identified a co‐localised cell population of CD4 and Granzyme B (GzmB), defined as CD4^+^ CTLs (**Figure** **1**A). The results showed that osteoporotic samples exhibited significantly fewer CD4^+^ CTLs than those of normal bone tissue samples (Figure 1B). Subsequently, we determined the ratio of CD4^+^ CTLs to total CD4^+^ T cells in the bone tissue samples. We observed that the percentage of normal bone tissue samples was notably higher (56.53%) than that of osteoporotic samples (20.44%) (Figure 1C). We then performed immunofluorescence (IF) staining on the abovementioned bone tissue samples from older adults. Compared with osteoporosis‐derived bone tissue samples, we further found that normal bone tissue samples harbored a significantly greater number of CD4^+^ CTLs in close vicinity to osteoblasts (Figure 1D,E).

To identify whether the distribution of immune cell subsets in bone microenvironment is associated with the accumulation of senescent osteoblasts, we further conducted spatial analysis of cell interaction pairs. The results revealed significant interactions between senescent osteoblasts and CD4^+^ CTLs in bone tissue samples (Figure 1F). Image analysis of the region of interest (ROI) during IMC of the abovementioned bone tissue further revealed a significantly higher proportion of p16^INK4a+^ senescent osteoblasts in osteoporotic samples than that of normal human bone tissue (Figure 1G,H). Uniform Manifold Approximation and Projection (UMAP) dimensionality reduction revealed distinct clusters of various cell types in the bone microenvironment (Figure S1B, Supporting Information). Notably, there was a higher overlap between the osteoblast population and the senescence marker p16^INK4a^ (Figure S1C, Supporting Information). The sirtuin family has been widely demonstrated to have anti‐aging effects. Subsequently, we discovered that the expression of the sirtuin family was significantly associated with the immune cell infiltration level in senescent tissues; however, significant heterogeneity was discerned (Figure S1D, Supporting Information). By analysing single‐cell sequencing data from different senescent tissues, we ascertained that SIRT1 within the sirtuin family was significantly and positively correlated with CD4^+^ CTLs (Figure S1E, Supporting Information). After analysing single‐cell data from bone marrow, SIRT1 was similarly found to be significantly positively correlated with CD4^+^ CTLs (Figure 1I). Additionally, IF staining indicated the downregulation of SIRT1, a crucial protein involved in anti‐ageing and immune regulation, in osteoporotic samples (Figure S1F, Supporting Information).^[^ ^19^, ^20^ ^]^ In conclusion, there was a significant difference in the distribution of CD4^+^ CTLs between normal and osteoporotic bone samples that was closely associated with the degree of osteoblast senescence. However, further investigations are required to determine whether SIRT1 expression is pivotal in this process.

### 2.2 SIRT1 in Osteoblasts Affects the Distribution of CD4^+^ CTLs in the Bone Microenvironment

**Figure 2:**
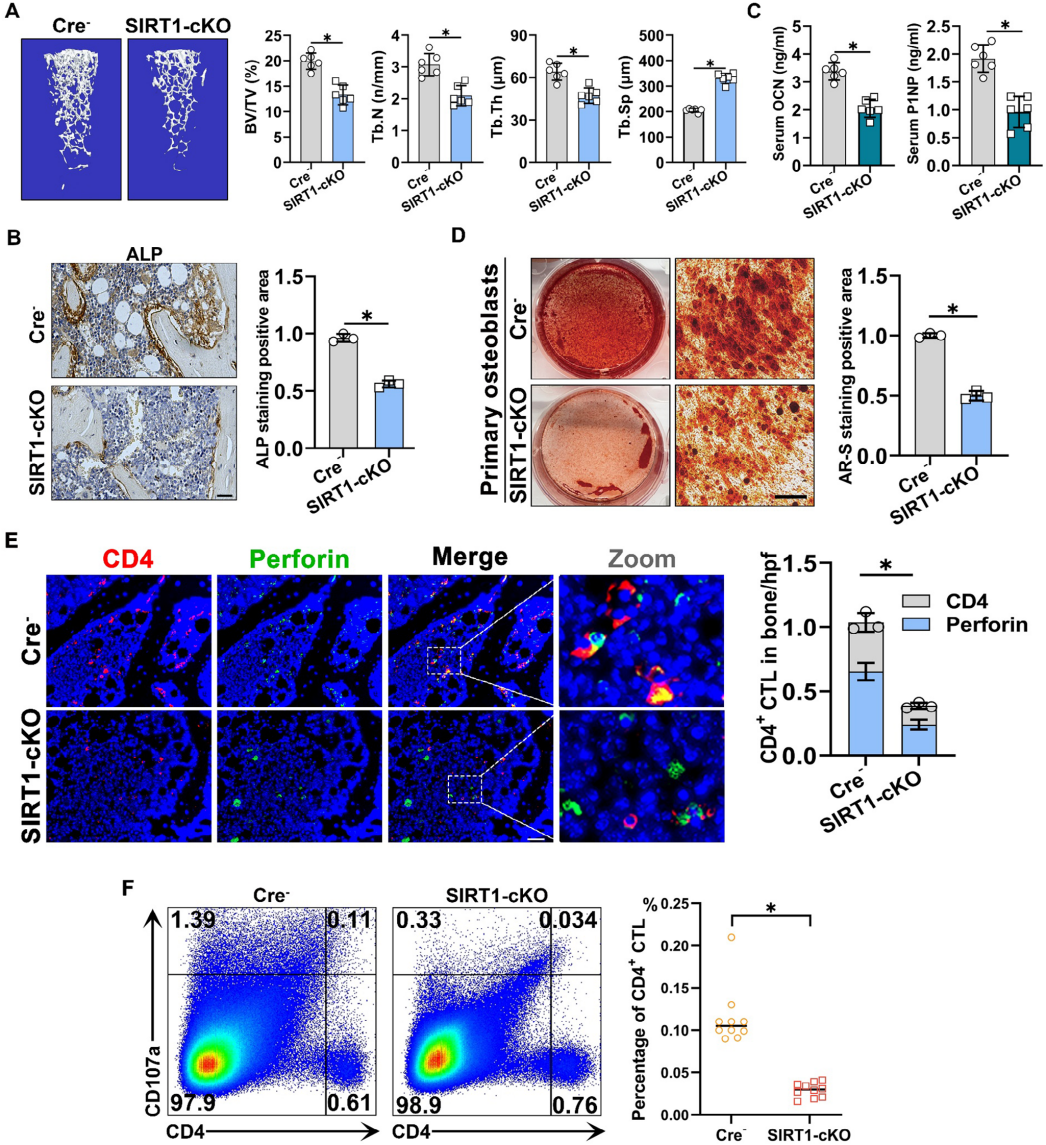
**SIRT1 in osteoblasts affects the distribution of CD4^+^ CTLs in the bone microenvironment A**) Representative Micro‐CT images and quantitative analyses of distal femora in male mice (*n* = 6). **B**) Representative images of IHC staining for detecting the ALP expression and quantitative analyses of ALP staining positive area (scale bar, 50 µm) (*n* = 3). **C**) Serum ELISA assays for bone formation markers (OCN and P1NP) (*n* = 6). **D**) Alizarin red (AR‐S) staining following osteogenic medium incubation for 21 days and quantitative analyses of AR‐S staining positive area (scale bar, 50 µm) (*n* = 3). **E**) Representative IF staining and quantitative analyses of CD4 and Perforin in Cre^−^ and SIRT1‐cKO mice bone samples. CD4^+^ cells are CD3^+^ *T* cells (scale bar, 50 µm) (*n* = 6). **F**) The proportion of CD4^hi^CD107a^hi^ CD4^+^ CTLs analysed using flow cytometry. Data are compared with the control group as the mean ± SD. Statistical significance: ^*^ *p* < 0.05.

To explore the immune mechanisms underlying OP caused by SIRT1 deficiency in osteoblasts, we generated osteoblast‐specific SIRT1‐knockout (cKO) mice by crossing SIRT1‐loxP with OC‐Cre mice. Western blotting results showed that the expression of SIRT1 protein was significantly decreased in SIRT1‐cKO mice. This indicates that SIRT1 has been successfully knocked out in osteoblasts (Figure S2A, Supporting Information). Micro‐CT analysis revealed that male SIRT1‐cKO mice at different months of age exhibited bone loss compared to age‐matched Cre^−^ control mice (Figure S2B, Supporting Information). Considering that the ovariectomized (Ovx) mouse model in subsequent experiments was established at 2 months of age with a 2‐month modeling period, we selected 4‐month‐old male SIRT1‐cKO mice to ensure consistent intervention time points between SIRT1‐deficient and Ovx groups in subsequent animal studies. Micro‐CT analysis revealed that in SIRT1‐cKO mice, the bone volume fraction (BV/TV), trabecular number (Tb.N), and trabecular thickness (Tb.Th) were reduced, whereas the trabecular spacing (Tb.Sp) increased, indicating the development of typical OP symptoms within 4 months post‐birth (**Figure** **2**A). Immunohistochemical (IHC) analysis of bone tissue sections revealed a significantly lower number of osteoblasts in SIRT1‐cKO mice than of WT mice (Figure 2B). Additionally, tartrate‐resistant acid phosphatase (TRAP) staining indicated no noticeable differences in osteoclast numbers on the bone trabecular surfaces between SIRT1‐cKO and WT mice (Figure S3A, Supporting Information). We further analysed serum markers of bone formation and resorption in SIRT1‐cKO and WT mice using an enzyme‐linked immunosorbent assay (ELISA) (Figures 2C; Figure S3B, Supporting Information). The results showed a decrease in bone formation markers [osteocalcin (OCN) and procollagen type I N‐terminal propeptide (P1NP)] and no significant change in bone resorption biomarkers [C‐terminal telopeptide of type I collagen (CTX‐1) and TRAP] in SIRT1‐cKO mice compared to those of WT mice. qRT‐PCR assays revealed significant downregulation of the osteogenesis‐related marker genes Runt‐related transcription factor 2 (RUNX2), collagen type I alpha 1 chain (COL1a1), OCN, osterix (OSX), and alkaline phosphatase (ALP) in SIRT1‐cKO mouse bone tissue compared to WT mice (Figure S3C, Supporting Information). However, the osteoclast‐related marker genes, TRAP MMP9, and CTSK, showed no significant differences (Figure S3D, Supporting Information). Western blotting results displayed the same trend as the qRT‐PCR findings (Figure S3E,F, Supporting Information). Subsequently, we cultured primary osteoblasts from 7‐day‐old mice in an osteogenic induction medium for 21 days. Alizarin red staining indicated a significant reduction in osteoblast mineralisation capacity in SIRT1‐cKO mice compared to that of WT mice (Figure 2D). To determine the changes in CD4^+^ CTL distribution in the bone microenvironment, we identified co‐localised CD4^+^ and perforin cell populations as CD4^+^ CTLs. IF staining revealed a reduced number of CD4^+^ CTLs near the bone trabeculae in SIRT1‐cKO mice compared to WT mice (Figure 2E; Figure S2C, Supporting Information). Flow cytometry showed that the number of CD4^hi^CD107a^hi^ CD4^+^ CTLs in the bone marrow of SIRT1‐cKO mice was significantly lower than that of WT mice (Figure 2F). Collectively, these findings suggest that SIRT1 deficiency in osteoblasts leads to an osteoporotic phenotype and substantial suppression of CD4^+^ CTLs in the vicinity of osteoblasts.

### 2.3 CD4^+^ CTLs Contribute to the Amelioration of OP by Eliminating Senescent Osteoblasts

**Figure 3:**
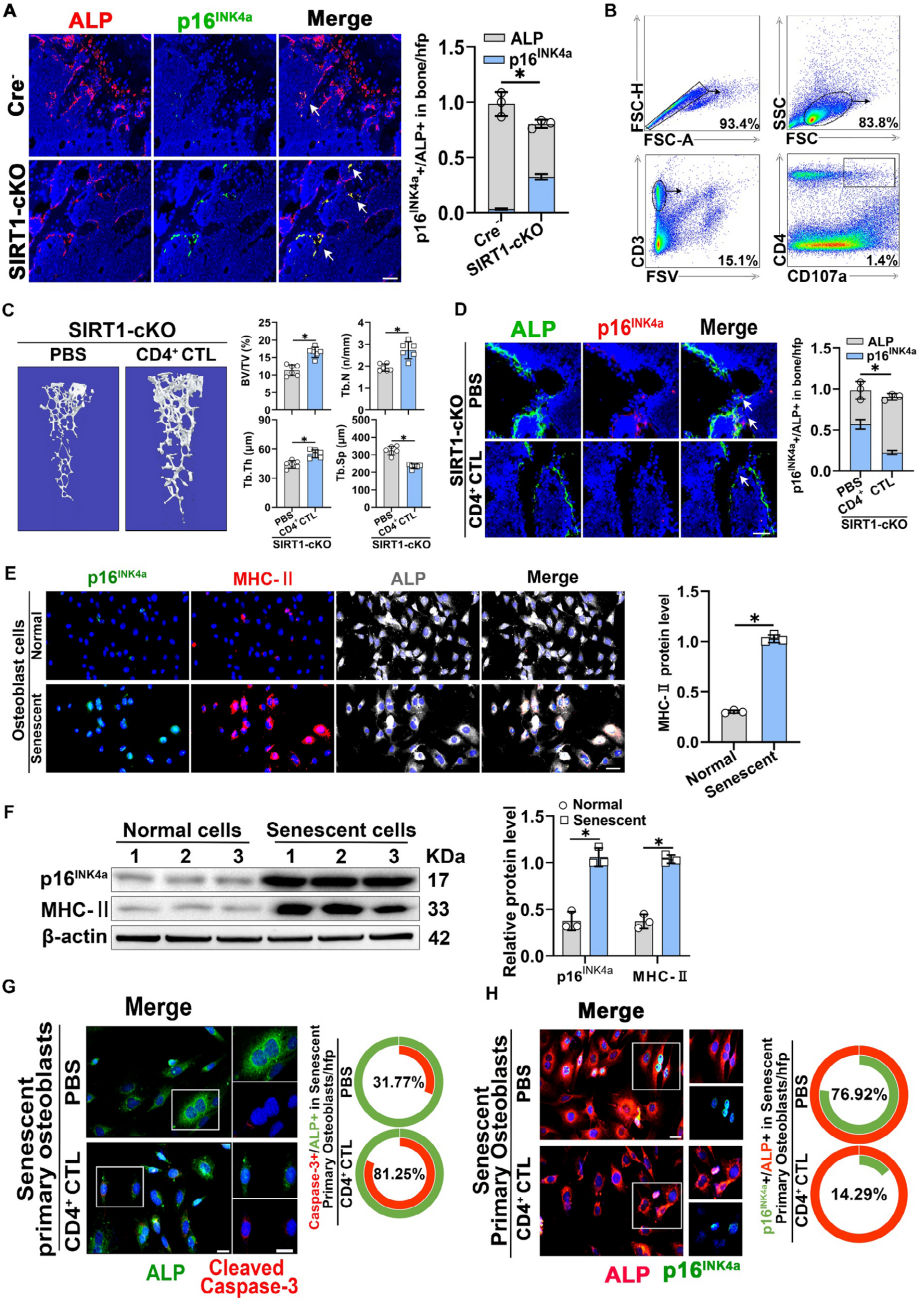
**CD4^+^ CTLs contribute to the amelioration of OP by eliminating senescent osteoblasts A**) Representative IF staining and quantitative analyses of ALP and p16^INK4a^ in Cre^−^ and SIRT1‐cKO mice bone samples (scale bar, 50 µm) (*n* = 3). **B**) CD3^hi^ CD4^hi^ CD107a^hi^ CD4^+^ CTLs isolated from spleen lymphocytes using flow cytometry. **C**) Representative Micro‐CT images and quantitative analyses of distal femora in SIRT1‐cKO mice after receiving tail vein CD4^+^ CTL injections for 6 weeks (*n* = 6). **D**) Representative images and quantitative analyses of the double staining of ALP and p16^INK4a^ in SIRT1‐cKO mice treated with CD4^+^ CTL injections for 6 weeks (scale bar, 50 µm) (*n* = 3). **E**) Representative IF staining of p16^INK4a^, MHC‐II, and ALP in normal and senescent primary osteoblasts and its quantification (scale bar, 50 µm) (*n* = 3). **F**) Representative western blots of p16^INK4a^ and MHC‐II expression in normal and senescent primary osteoblasts and their quantification (*n* = 3). **G**) Representative IF staining of cleaved caspase‐3 and ALP in senescent primary osteoblasts after co‐culture with CD4^+^ CTLs (scale bar, 50 µm). **H**) Representative IF staining of p16^INK4a^ and ALP in senescent primary osteoblasts after co‐culture with CD4^+^ CTLs (scale bar, 50 µm). Data are compared with the control group as the mean ± SD. Statistical significance: ^*^ *p* < 0.05.

Although CD8^+^ T cells are known for their cytotoxic activity, there is increasing evidence of cytotoxicity in CD4^+^ T cells, primarily dependent on perforin.^[^ ^21^, ^22^ ^]^ Particularly in the elderly, the response of CD4^+^ CTLs is associated with in vivo elimination of senescent cells.^[^ ^10^, ^13^ ^]^ IF staining revealed a higher number of senescent osteoblasts in the bone tissues of SIRT1‐cKO mice than that of WT mice (**Figure** **3**A). These cells were mainly found at the periphery of the bone trabeculae, suggesting a strong connection between OP in SIRT1‐deficient bone tissues and the overaccumulation of senescent osteoblasts. To determine whether the SIRT1 deletion in osteoblasts leading to OP correlates with decreased CD4^+^ CTLs in the bone microenvironment, we extracted lymphocytes from littermate spleens and isolated CD3^hi^ CD4^hi^ CD107a^hi^ CD4^+^ CTLs using flow cytometry (Figure 3B). These cells were cultured in IL‐2‐enriched medium. Subsequently, SIRT1‐cKO mice were divided into a sham‐operated control group and another receiving tail vein injections of 5×10^6^ CD4^+^ CTLs bidaily (Figure S4A, Supporting Information). After 6 weeks, Micro‐CT analysis revealed increased BV/TV, Tb.N, and Tb.Th, and decreased Tb.Sp in the CD4^+^ CTL‐injected group compared to those of the control, suggesting effective mitigation of OP in SIRT1‐cKO mice (Figure 3C). We conducted qRT‐PCR assays and western blotting to verify the CD4^+^ CTL injections. Both qRT‐PCR and western blotting results indicated a substantial increase in osteogenesis‐related marker genes and proteins in the bone tissues of the CD4^+^ CTL‐injected group compared to the control (Figure S4B,C, Supporting Information). In addition, by performing a stress test on the cortical bone of mice injected with CD4^+^ CTLs, we found that the cortical bone of mice supplemented with CD4^+^ CTLs had better compressive properties than that of the control group (Figure S4D, Supporting Information). IF staining indicated a notable decrease in the number of senescent osteoblasts in the bone tissues of CD4^+^ CTL‐injected mice, particularly around the bone trabeculae (Figure 3D).

MHC‐II surface expression is critical for CD4^+^ CTL‐elicited immunity. To determine whether senescent osteoblasts could be a direct target of CD4^+^ CTL, we induced senescence of primary mouse osteoblasts using D‐galactose (Figure S4E, Supporting Information). Thereafter, we examined the expression of MHC‐II on senescent primary osteoblasts through IF staining and western blotting (Figure 3E,F). Importantly, we found that MHC‐II was highly expressed in senescent osteoblasts while its expression was negligible in normal osteoblasts. These findings support the involvement of the CD4^+^ CTL/MHC‐II axis in the immunosurveillance of senescent osteoblasts. Next, we assessed whether CD4^+^ CTLs specifically target and eliminate senescent osteoblasts. We co‐cultured senescent primary mouse osteoblasts with CD4^+^ CTL (Figure S4F, Supporting Information). IF staining revealed a higher proportion of cleaved caspase‐3^+^ apoptotic osteoblasts in the co‐culture with CD4^+^ CTLs than that of the control group (Figure 3G). IF staining results also showed that the proportion of senescent osteoblasts among the total osteoblasts co‐cultured with CD4^+^ CTLs was much lower than that in the control group (Figure 3H). These results imply that CD4^+^ CTLs can directly recognise and eliminate senescent osteoblasts, which are highly immunogenic in the bone tissue, thereby reducing the accumulation of senescent osteoblasts and ameliorating the progression of OP.

### 2.4 DAAM2 Downstream of SIRT1 Regulates Chemokine Secretion

**Figure 4:**
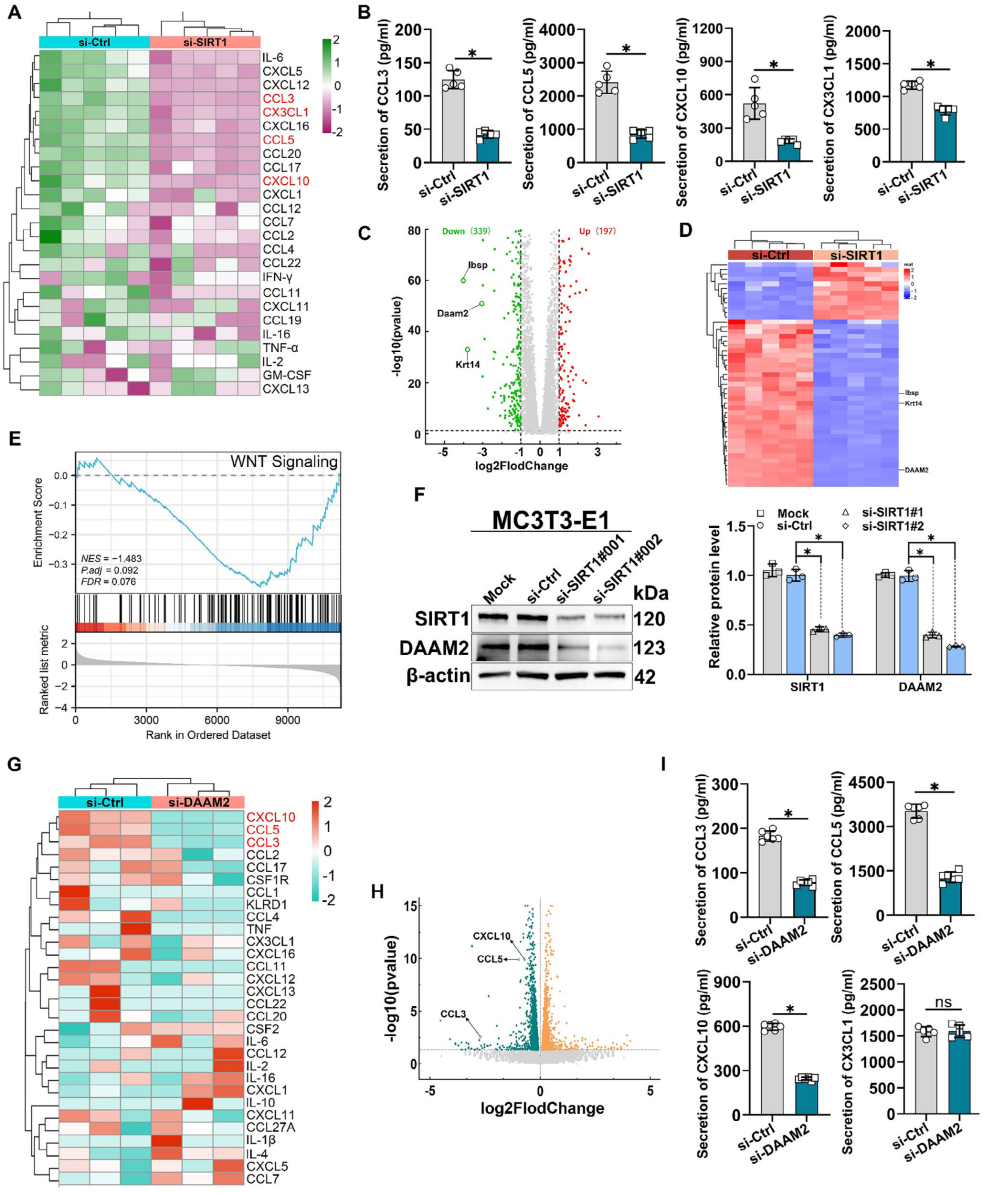
**DAAM2 downstream of SIRT1 regulates chemokine secretion A**) Heatmap showing the secretion of chemokines enriched in MC3T3‐E1 cell culture supernatant. The colour indicates fold change in chemokine levels determined via Luminex liquid suspension chip (*n* = 5). **B**) Secretion of CCL3, CCL5, CXCL10, and CX3CL1 in MC3T3‐E1 cells culture supernatant determined via Luminex liquid suspension chip. **C**) Volcano plot of RNA‐seq data displaying the gene expression pattern in the post‐si‐SIRT1 intervention versus the si‐Ctrl group. Significantly downregulated osteoporosis‐related genes are highlighted. **D**) Cluster analysis of the differentially expressed genes. **E**) GSEA of the differentially expressed genes (*n* = 5). **F**) Representative western blots of SIRT1 and DAAM2 expression in MC3T3‐E1 cells and quantification (*n* = 3). **G**) RNA‐seq showing the levels of chemokines enriched in the post‐si‐DAAM2 intervention group versus the si‐Ctrl group. **H**) Volcano plot of RNA‐seq data displaying the gene expression pattern in the post‐si‐DAAM2 intervention versus the si‐Ctrl group. Significantly downregulated chemokines are highlighted. **I**) Secretion of CCL3, CCL5, CXCL10, and CX3CL1 in MC3T3‐E1 cells culture supernatant determined via ELISA assays (*n* = 6). Data are presented compared with the control group as the mean ± SD. Statistical significance: ^*^ *p* < 0.05.

Previous studies have mainly focused on the cell‐autonomous role of SIRT1 in immune regulation, but it is currently unclear how SIRT1 in osteoblasts affects the distribution of CD4^+^ CTLs. Initially, si‐SIRT1 was applied to MC3T3‐E1 cells, the precursor of mouse embryonic osteoblasts, for 36 h, followed by a shift to a serum‐free medium for another 36 h incubation. Chemokine levels in the cell supernatant were measured using Luminex‐based antibody microarray technology (**Figure** **4**A), with emphasis on the CD4^+^ CTLs’ chemokine receptors (CCR5, CXCR3, and CX3CR1).^[^ ^23^, ^24^ ^]^ The assay results indicated a significant reduction (approximately two‐fold or more) in CCL3, CCL5, and CXCL10 levels and a modest decrease in CX3CL1 in the supernatants of the si‐SIRT1 group compared to the si‐Ctrl group (Figure 4B).

To identify genes downstream of SIRT1 that regulate chemokine secretion, we performed si‐SIRT1 intervention and RNA‐seq analysis on MC3T3‐E1 cells. Overall, 536 differentially expressed genes were identified, including 197 and 339 up‐ and down‐regulated genes, respectively (Figure 4C). Our cluster analysis of these differentially expressed genes (|log2 fold change (FC)| > 2) revealed that DAAM2, a key regulator of the Wnt signalling pathway and identified as a major gene associated with OP susceptibility in a large sample analysis, exhibited significant downregulation (Figure 4D).^[^ ^25^, ^26^, ^27^ ^]^ Notably, DAAM2 showed one of the most prominent downregulations among all differentially expressed genes (log2FC = −3.0) and ranked in the top three, making it a standout target in our screening. Further, our correlation analysis via Gene Expression Profiling Interactive Analysis 2 (GEPIA2) demonstrated a strong positive association between SIRT1 and DAAM2 expression (Figure S5A, Supporting Information), reinforcing its potential as a downstream effector of SIRT1.

To explore a role for SIRT1 in OP, we conducted Gene Set Enrichment Analysis (GESA) of SIRT1‐associated genes, and our results revealed that SIRT1 was involved in the Wnt signaling pathway — a pathway critical for osteoblast differentiation and survival (Figure 4E). Following si‐SIRT1 intervention in MC3T3‐E1 cells, western blotting revealed reduced DAAM2 expression in the si‐SIRT1 groups compared to the mock and si‐Ctrl groups, suggesting SIRT1's positive regulation of DAAM2 protein expression (Figure 4F). Given the prior evidence from large‐scale genetic studies linking DAAM2 polymorphisms to osteoporosis susceptibility,^[^ ^26^ ^]^ DAAM2 logically emerged as a prioritized mediator linking SIRT1 to immune regulation in the bone microenvironment. Subsequently, RNA‐seq analysis post‐si‐DAAM2 intervention in MC3T3‐E1 cells indicated significant decreases in CCL3, CCL5, and CXCL10 levels compared to those in the si‐Ctrl group (Figure 4G,H). ELISA was used to measure the concentrations of these chemokines in cell supernatants, confirming the consistency of the sequencing results (Figure 4I). This led us to hypothesise that DAAM2 is a crucial target for SIRT1's downstream regulation of CCL3, CCL5, and CXCL10.

### 2.5 SIRT1 Modulates CD4^+^ CTLs via the EZH2/DAAM2/Chemokine Axis

**Figure 5:**
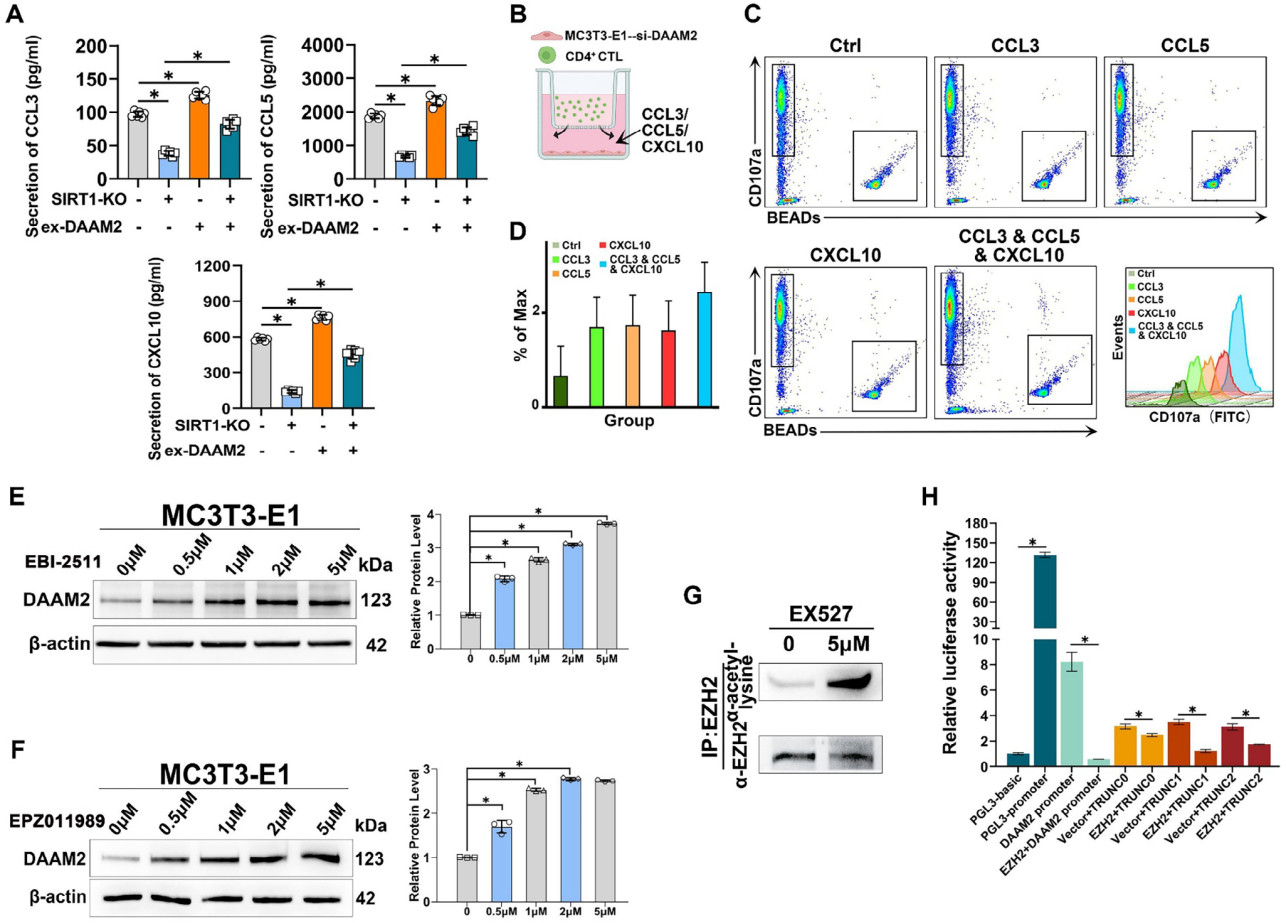
**SIRT1 modulates CD4^+^ CTLs via the EZH2/DAAM2/chemokine axis A**) The secretion of CCL3, CCL5, and CXCL10 in the culture supernatant of WT and SIRT1‐KO MC3T3‐E1 cells overexpressing DAAM2 was determined via ELISA assays (*n* = 6). **B**) Transwell co‐culture example figure. **C**) The number of cells passing through the lower Transwell chamber was calculated using flow cytometry. **D**) Mean fluorescence intensity of CD107a. **E,F**) Representative western blots of the expression of DAAM2 protein and its quantification in MC3T3‐E1 cells treated with different concentrations of EZH2 inhibitors EBI‐2511 and EPZ011989, respectively. **G**) EZH2 acetylation levels were measured using western blots after immunoprecipitation of MC3T3‐E1 cell lysates treated with SIRT1 inhibitor EX527. **H**) Relative luciferase activity analysis of TRUNC0, TRUNC1, and TRUNC2 (*n* = 3). Data are presented compared with the control group as the mean ± SD. Statistical analysis: ^*^ *p* < 0.05.

To further validate the precise role of DAAM2 downstream of SIRT1, we created a SIRT1‐KO MC3T3‐E1 cell line (Figure S5B, Supporting Information) and overexpressed DAAM2 using a lentivirus. ELISA of cell supernatants revealed significantly lower levels of CCL3, CCL5, and CXCL10 in the SIRT1‐KO group than those in the WT group (**Figure** **5**A). Notably, DAAM2 overexpression reversed this downregulation. We investigated the effects of DAAM2 and these chemokines on CD4^+^ CTLs using Transwell co‐culture experiments (Figure 5B). MC3T3‐E1 cells treated with si‐DAAM2 were placed in the lower chamber of a Transwell with a chemokine‐containing basal medium. CD4^+^ CTLs were co‐cultured in the upper chamber. After 2 h, flow cytometry was used to count the suspended CD4^+^ CTLs expressing CD107a on their surfaces in the lower chamber. CD4^+^ CTL counts were increased in the groups treated with CCL3, CCL5, and CXCL10, suggesting that these chemokines are crucial for enhancing cellular migration (Figure 5C). Adding these chemokines increased CD107a expression in CD4^+^ CTLs to varying degrees, indicating enhanced degranulation activity (Figure 5D). These data imply that DAAM2, a downstream target of SIRT1, plays a crucial role in regulating CCL3, CCL5, and CXCL10 secretion, positively influencing CD4^+^ CTLs migration and activation.

To elucidate the molecular mechanism by which SIRT1 regulates DAAM2 expression, we screened small‐molecule inhibitors targeting key proteins downstream of SIRT1 (Figure S5C–F, Supporting Information). Following the separate application of two EZH2 inhibitors (EBI‐2511 and EPZ011989) to MC3T3‐E1 cells, western blotting revealed a significant upregulation of DAAM2 expression in the inhibitor‐treated groups compared to that in the controls, with a positive correlation between DAAM2 expression and EZH2 inhibitor concentration (Figure 5E,F). Next, we immunoprecipitated lysates from MC3T3‐E1 cells treated with the SIRT1 inhibitor, EX527, using an EZH2 antibody. Western blot analysis indicated a significant increase in EZH2 acetylation in cells treated with the SIRT1 inhibitor compared to that in phosphate‐buffered saline (PBS) controls, implying that SIRT1 directly regulates EZH2 acetylation and histone methyltransferase activity in MC3T3‐E1 cells (Figure 5G). To confirm whether EZH2 modulates DAAM2 expression by inhibiting *DAAM2* transcription, we conducted a luciferase reporter gene assay (Figure 5H). We engineered pGL3 vectors (TRUNC0, TRUNC1, and TRUNC2) containing the DAAM2 promoter and truncated EZH2 binding regions. Cells were transfected with EZH2 overexpression vectors, using pGL3‐basic as a control. Luciferase assay results demonstrated that EZH2 binds to the DAAM2 promoter and represses its transcription, dependent on the promoter segment truncated by TRUNC1. These findings suggested that SIRT1 modulates CD4^+^ CTL migration and activation in the bone microenvironment via the EZH2/DAAM2/chemokine (CCL3, CCL5, and CXCL10) axis.

### 2.6 Overexpression of DAAM2 Rescues the Osteoporotic Bone Loss Caused by SIRT1 Deficiency in Osteoblasts by Enhancing CD4^+^ CTL Responses

**Figure 6:**
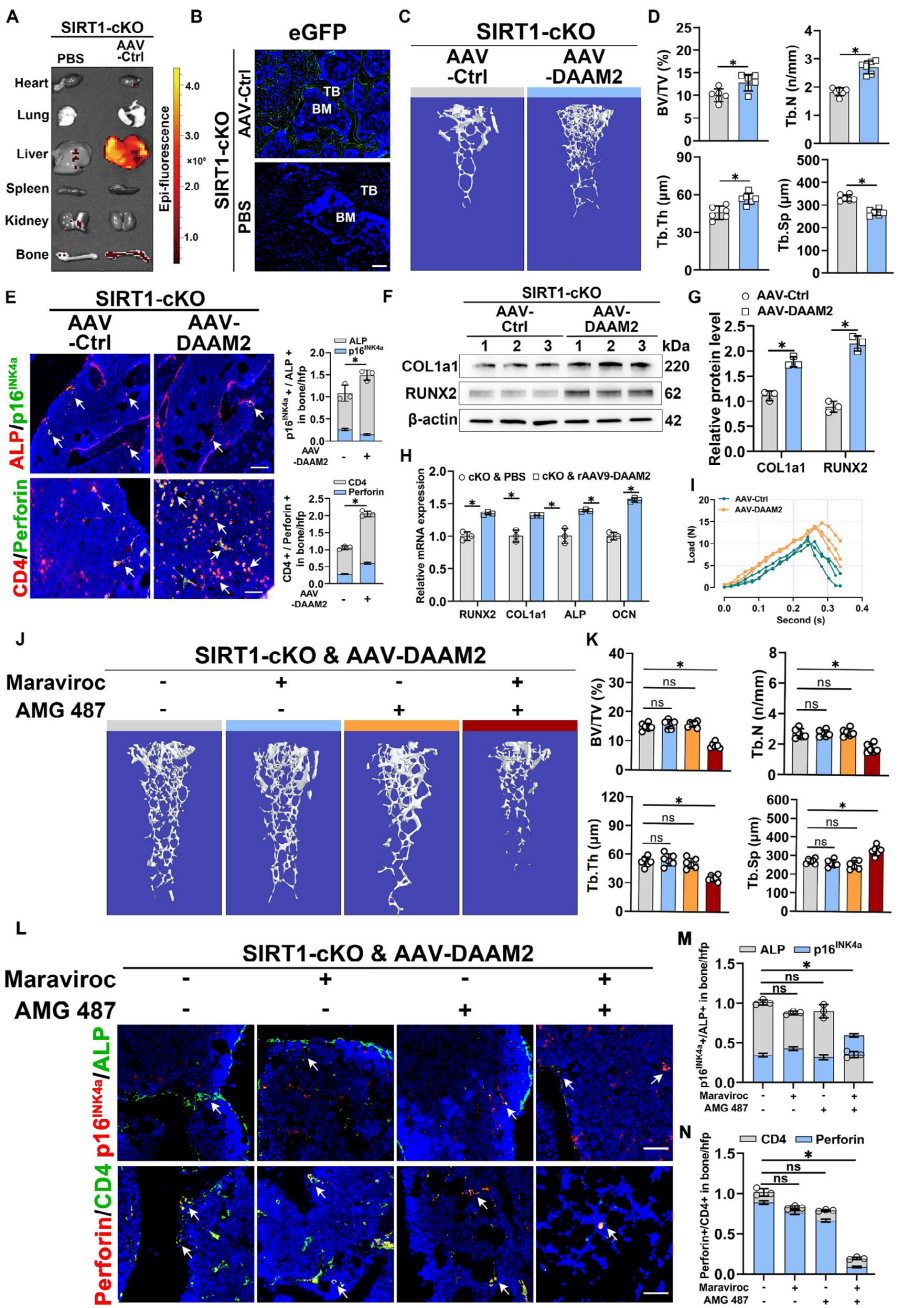
**Overexpression of DAAM2 rescues osteoporotic bone loss caused by SIRT1 deficiency in osteoblasts through enhancing CD4^+^ CTL responses A**) AAV vectors were intravenously (i.v.) injected into 8‐week‐old mice, and eGFP expression in individual tissues was monitored via IVIS‐100 optical imaging 2 weeks post‐injection. **B**) eGFP expression was assessed using fluorescence microscopy on cryo‐sectioned femur (scale bar, 50 µm). **C**,**D**) Representative Micro‐CT images and quantitative analyses of distal femora in mice (*n* = 6). **E**) Representative images and quantitative analyses of the double staining of ALP and p16^INK4a^, as well as CD4 and Perforin in distal femora in mice (scale bar, 50 µm) (*n* = 3). **F**,**G**) Representative western blots of COL1a1 and RUNX2 expression in bone samples of mice and their quantification (*n* = 3). **H**) qRT‐PCR of the relative osteoblast marker gene (RUNX2, Col1a1, OCN, and ALP) mRNA levels of the groups indicated (*n* = 3). **I**) Stress test used to detect the femoral cortex to withstand stress (*n* = 3). **J**,**K**) Representative Micro‐CT images and quantitative analyses of distal femora in SIRT1‐cKO mice injected with AAV‐DAAM2 into four groups: PBS, CCR5 antagonist (Maraviroc), CXCR3 antagonist (AMG 487), and mixed antagonists (Maraviroc & AMG 487). (*n* = 6). **L**–**N**), Representative images and quantitative analyses of the double staining of ALP and p16^INK4a^, as well as CD4 and Perforin in distal femora in mice (scale bar, 50 µm) (*n* = 3). Data are compared with the control group as the mean ± SD. Statistical significance: ^*^ *p* < 0.05.

To elucidate the role of DAAM2, downstream of SIRT1 in treating OP, we developed bone‐targeted adeno‐associated virus (AAV) vectors incorporating DAAM2 (AAV‐DAAM2) and enhanced osteoblast targeting by inserting a DSS‐encoded DNA sequence into the AAV vectors.^[^ ^28^ ^]^ Initially, we injected 2‐month‐old mice with empty AAV vectors encoding eGFP (AAV‐Ctrl) and 2 weeks later assessed the tissue distribution of AAV vectors by measuring luciferase bioluminescence intensity. Organ imaging revealed that AAV vectors were primarily expressed in the bone tissue, with notable enrichment in the liver tissue, owing to their naturally high affinity (**Figure** **6**A). IF staining of frozen bone tissue sections indicated predominant eGFP expression in the trabecular region (Figure 6B). These findings imply that systemically administered AAV vectors can effectively target osteoblast‐lineage cells.

For the in vivo study of DAAM2 effects on bone salvage and CD4^+^ CTL distribution in osteoblast‐specific SIRT1 knockout mice, we randomly assigned 2‐month‐old SIRT1‐cKO mice into two groups, each receiving different AAV vector injections. Micro‐CT analyses revealed that AAV‐DAAM2‐injected SIRT1‐cKO mice exhibited significantly higher BV/TV, Tb.N, and Tb.Th, and lower Tb.Sp compared to those of control mice receiving AAV‐Ctrl injections (Figure 6C,D). IHC analysis indicated a significantly higher number of osteoblasts in the bone tissues of AAV‐DAAM2‐injected mice than those of controls (Figure S6A, Supporting Information). Both qRT‐PCR and western blot analyses showed a significant upregulation of osteogenesis‐related genes and proteins following DAAM2 overexpression in vivo (Figure 6F–H). IF staining co‐localisation analysis revealed a decrease in senescent osteoblasts and an increase in CD4^+^ CTLs around the bone trabeculae in AAV‐DAAM2‐injected mice compared to controls (Figure 6E). In addition, by performing a stress test on the cortical bone of mice injected with AAV‐DAAM2, we found that the cortical bone of mice overexpressing DAAM2 had better compressive properties than that of the control group (Figure 6I). This implies that DAAM2 regulates CD4^+^ CTL distribution and contributes to the removal of senescent osteoblasts, thereby influencing OP.

To study the in vivo effects of DAAM2‐regulated chemokines (CCL3, CCL5, and CXCL10) on bone mass and CD4^+^ CTL distribution, 2‐month‐old SIRT1‐cKO mice injected with AAV‐DAAM2 were divided into four groups: PBS, CCR5 antagonist (Maraviroc), CXCR3 antagonist (AMG 487), and mixed antagonists (Maraviroc and AMG 487). Micro‐CT analysis revealed no statistical differences in bone indices (BV/TV, Tb.N, Tb.Th, and Tb.Sp) between mice treated with CCR5 or CXCR3 antagonists and those treated with PBS. However, the group receiving the mixed antagonists exhibited significantly decreased bone mass (Figure 6J,K). IHC analysis indicated a significant reduction in osteoblasts in the mixed antagonist group compared to the PBS group. In contrast, no notable change was observed in the groups receiving single antagonists (Figure S6D, Supporting Information). Western blotting results corroborated these findings (Figure S6B,C, Supporting Information). Notably, IF staining analysis showed an increase in senescent cells and a decrease in CD4^+^ CTLs aggregates around the bone trabeculae in the mixed antagonist group compared to the other groups (Figures 6L–N). In conclusion, DAAM2 overexpression in vivo enhanced CD4^+^ CTL distribution and mitigated bone loss caused by SIRT1 deficiency in osteoblasts, which was abrogated when the binding of chemokines (CCL3, CCL5, and CXCL10) to CD4^+^ CTLs’ chemokine receptors was fully inhibited.

### 2.7 Reduced SIRT1/DAAM2 Expression Levels and CD4^+^ CTL Numbers are Prevalent in the Osteoporotic Bone Microenvironment

**Figure 7:**
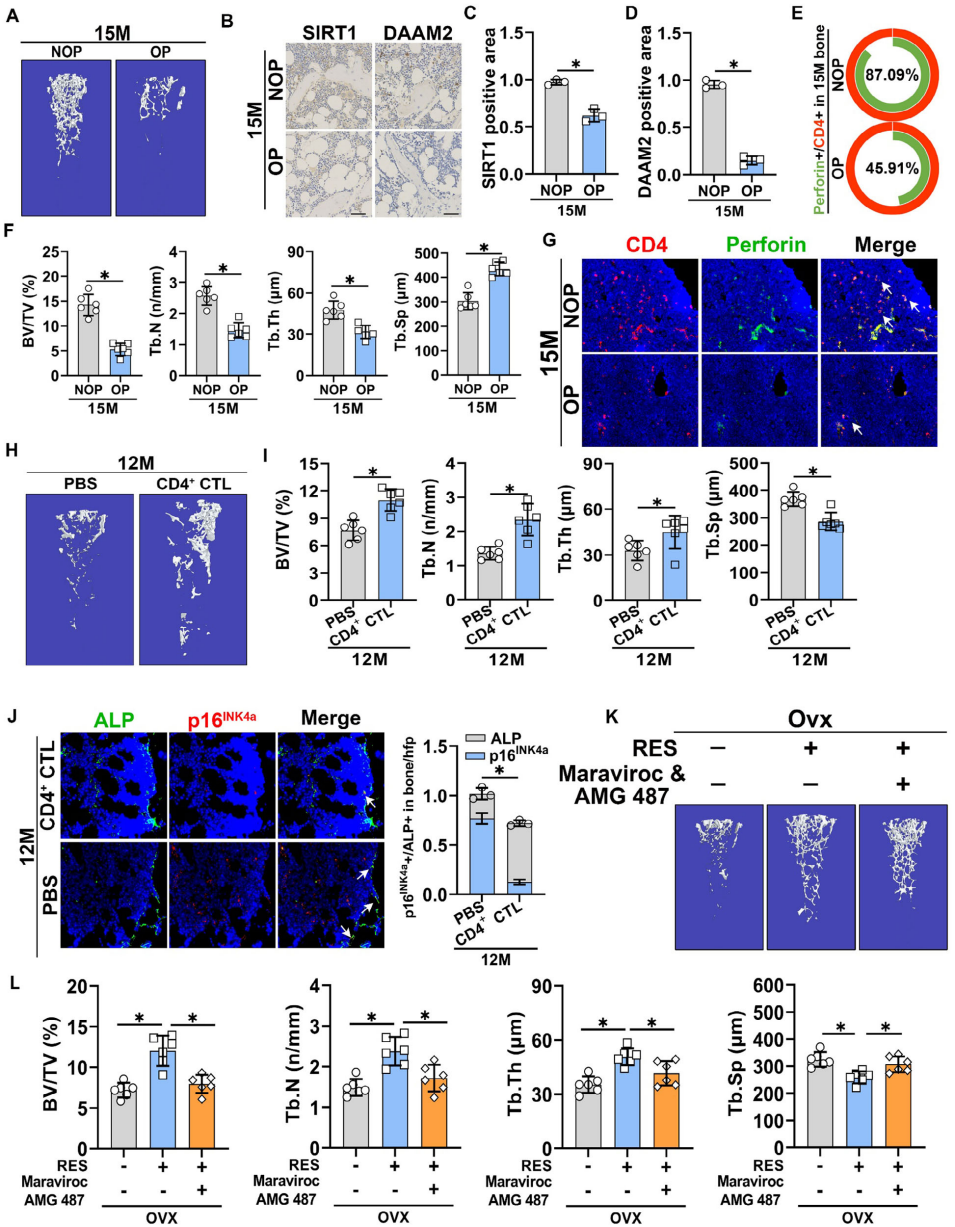
**Reduced SIRT1/DAAM2 expression levels and CD4^+^ CTL numbers are prevalent in the osteoporotic bone microenvironment A**) and **F**). Representative Micro‐CT images and quantitative analysis of distal femurs in 15‐month‐old non‐osteoporotic and osteoporotic mice. **B**–**D**), IHC staining and respective quantitative analysis of SIRT1 and DAAM2 on the bone surface (scale bar, 50 µm) (*n* = 3). **E**–**G**), Representative images and quantitative analyses of the double staining of CD4 and Perforin in distal femora in mice (scale bar, 50 µm). **H**,**I**) Representative Micro‐CT images and quantitative analyses of distal femora in 12‐month‐old mice with CD4^+^ CTLs injection for 6 weeks (*n* = 6). **J**) Representative images and quantitative analyses of the double staining of ALP and p16^INK4a^ in distal femora in mice (scale bar, 50 µm) (*n* = 3). **K**,**L**), Representative Micro‐CT images and quantitative analyses of distal femora in Ovx mice administered SIRT1 agonists resveratrol (RES) with or without concurrent Maraviroc & AMG 487 (*n* = 6). Data are compared with the control group as the mean ± SD. Statistical significance: ^*^ *p* < 0.05.

To determine whether changes in SIRT1, DAAM2, and CD4^+^ CTLs were consistent across various OP models, we conducted observational analyses of deovulated and aged OP mouse models. Micro‐CT analysis revealed typical osteoporotic changes in the castrated mouse (Ovx) group compared with those in the sham‐operated group (Figure S7A, Supporting Information). IHC and IF staining indicated a decrease in SIRT1 and DAAM2 expression and the number of CD4^+^ CTLs in the Ovx group compared with those in the sham group (Figure S7B,C, Supporting Information). Concurrently, we performed Micro‐CT analysis on the femurs of naturally aged 15‐month‐old mice, selecting six bone tissue samples from high and low bone mass mice, respectively (**Figure** **7**A,F). IHC and IF staining showed reduced SIRT1 and DAAM2 expression (Figure 7B–D) and fewer CD4^+^ CTLs in aged mice with low bone mass than those with high bone mass (Figure 7E,G). To investigate the impact of DAAM2 on OP, Ovx mice were randomized into two groups. Compared with the control group mice given AAV‐Ctrl, the mice administered adeno‐associated virus AAV‐DAAM2 showed a partial increase in bone mass (Figure S7D, Supporting Information). To demonstrate the direct impact of CD4^+^ CTLs on OP, we injected proliferation‐activated CD4^+^ CTLs into both the Ovx and 12‐month‐old groups. After six weeks, we observed increased BV/TV, Tb.N, and Tb.Th, and decreased Tb.Sp in these groups compared to the PBS‐injected controls (Figure 7H,I; Figure S7E, Supporting Information). IF staining revealed that CD4^+^ CTL injection notably reduced the number of senescent osteoblasts (Figure 7J; Figure S7F, Supporting Information). To validate whether exogenous supplementation of CD4^+^ CTLs could mitigate bone loss by modulating osteoclastic bone resorption in Ovx‐induced osteoporosis, we performed calcein double‐labeling experiments. Results showed that mineral apposition rate (MAR) and bone formation rate (BFR) were significantly decreased in Ovx mice compared to controls, and both parameters improved following CD4^+^ CTL supplementation (Figure S7G,H, Supporting Information). TRAP staining revealed a modest reduction in osteoclast number following CD4^+^ CTL treatment (Figure S7I,J, Supporting Information). Furthermore, ELISA analysis of serum biomarkers confirmed decreased levels of CTX‐1 and TRAP‐5b, indicating suppressed bone resorption activity (Figure S7K, Supporting Information). Collectively, these findings indicate that exogenous supplementation of CD4^+^ CTLs may additionally inhibit bone loss by regulating osteoclastic bone resorption in the Ovx‐induced osteoporosis model, which warrants further investigation in future studies.

Given that SIRT1 agonists mitigate OP, we investigated whether blocking SIRT1‐regulated chemokines (CCL3, CCL5, and CXCL10) that control CD4^+^ CTLs could inhibit this effect. Hence, we administered the SIRT1 agonist resveratrol (RES) to Ovx mice with or without concurrent Maraviroc and AMG 487 injections for 6 weeks. Micro‐CT analysis revealed that the bone mass decreased in mice treated with the combined mixed antagonist compared to those receiving only SIRT1 agonists (Figure 7K,L). These findings imply that reductions in SIRT1 and DAAM2 expression, often accompanied by decreased CD4^+^ CTLs, are characteristic of the osteoporotic bone microenvironment. Furthermore, the direct injection of CD4^+^ CTLs can effectively counter OP.

## 3 Discussion

**Figure 8:**
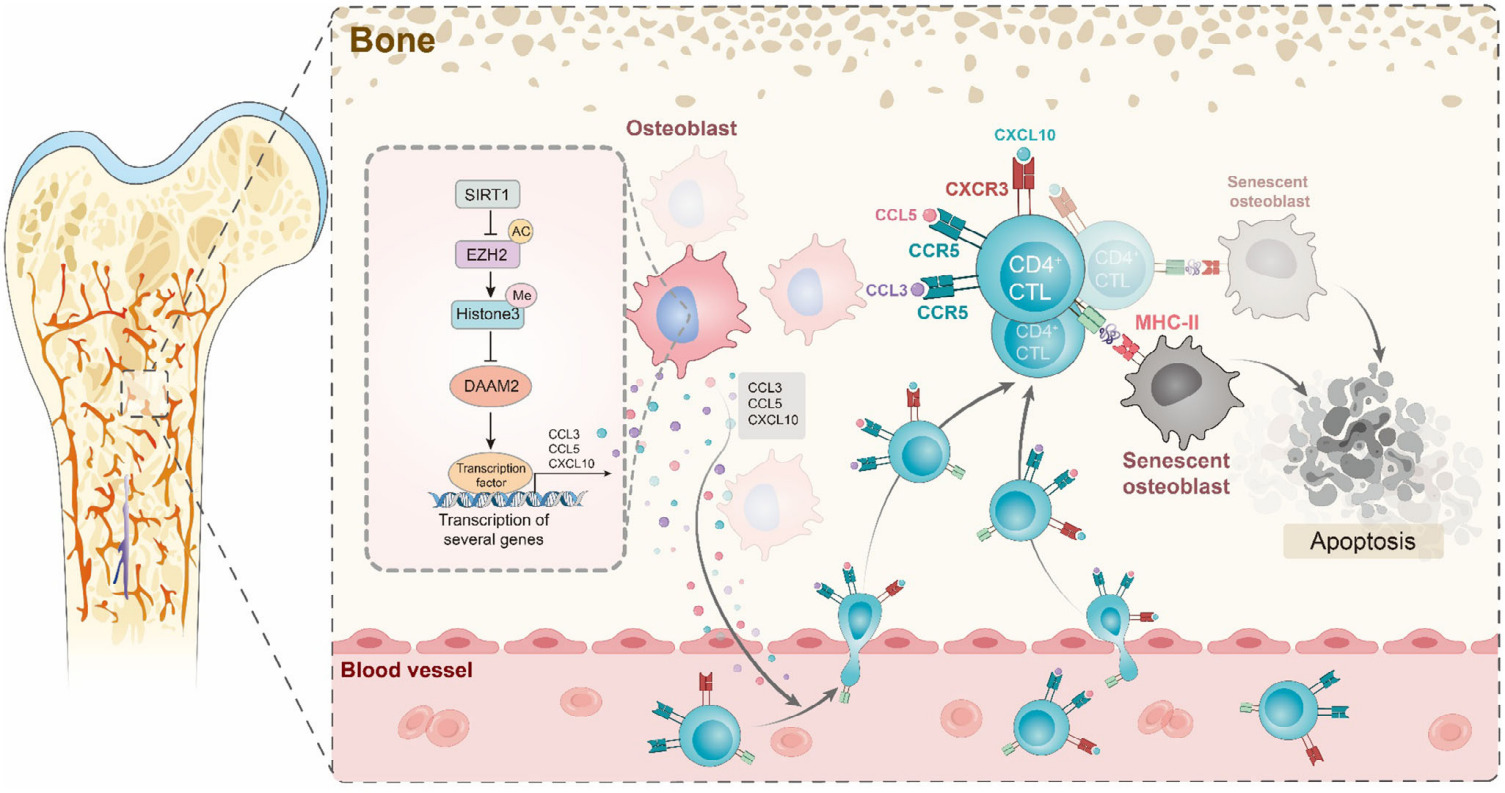
**SIRT1 orchestrating bone mass maintenance through involving osteoblast‐CD4^+^ CTL crosstalk**. In the osteoblastic niche, SIRT1 activates and recruits CD4^+^ CTLs by increasing DAAM2 expression via EZH2 deacetylation and boosting the secretion of key chemokines, such as CCL3, CCL5, and CXCL10. Then, CD4^+^ CTL directly eliminates senescent osteoblasts in an MHC‐II‐dependent way, thereby slowing down the process of bone ageing and effectively alleviating OP.

In this study, we show that beyond the well‐studied cell‐autonomous mechanisms of SIRT1, it also exerts a novel non‐cell‐autonomous function by regulating the osteoblast‐CD4^+^ CTL crosstalk. Specifically, in the osteoblastic niche, SIRT1 promotes the secretion of key chemokines (e.g., CCL3, CCL5, and CXCL10) by upregulating DAAM2 through acetylation of EZH2 protein, thereby activating and recruiting CD4^+^ CTLs. Then CD4^+^ CTLs eliminate senescent osteoblasts in an MHC‐II‐dependent manner, slowing the bone ageing process and effectively ameliorating OP (**Figure** **8**). Additionally, this study suggests that the impact of the immune system on bone diseases is more significant than was previously understood.

At first glance, the bone may appear just a hard substance that serves merely as a scaffold. As a highly active organ, bone also has diverse functions. Bone is a metabolic entity that can store minerals, the cradle of our defence system, a hormonal organ involved in glucose metabolism, a site of haematopoiesis, and a rich source of stem cells. Bone is, therefore, central to human health. The bone microenvironment, crucial for regulating bone metabolism and immunosurveillance, involves interactions between various cell types, including osteoblasts, osteoclasts, adipocytes, and immune cells.^[^ ^29^, ^30^ ^]^ These interactions profoundly affect bone development, formation, homeostasis, and disease.^[^ ^31^, ^32^ ^]^ The accumulation of senescent osteoblasts in the bone microenvironment can exacerbate bone loss.

Age‐related osteoblast dysfunction, a primary cause of bone loss in individuals over 50 years of age, stems from external changes in the bone microenvironment and internal senescence of osteoblasts.^[^ ^33^ ^]^ Accumulating evidence indicates that immune cells regulate bone homeostasis, leading to the term “osteoimmunology,” which describes the bidirectional interaction between the bone and the immune system.^[^ ^34^ ^]^ Hence, comparing immune cell distribution in physiologically “young” and “aged” individuals within the ageing population would probably elucidate bone ageing mechanisms from an immune standpoint. The key effector lymphocytes in the adaptive immune system are B, CD4^+^ T, and CD8^+^ T cells. The adaptive immune system weakens with ageing, and thymic atrophy reduces naïve T cell production.^[^ ^9^, ^35^ ^]^ Immune senescence is typically marked by significant changes in the adaptive immune system with age. An analysis of immune function in supercentenarians showed a proportion of CD4^+^ CTLs (≈25.3% of total T cells) higher than that of young controls (≈2.8%). However, the total T‐cell count was similar in both groups.^[^ ^13^ ^]^ To determine whether these immune cell distribution changes also occur in the bone microenvironment, we conducted IMC on bone tissue samples from men aged > 60 years. We observed higher SIRT1 protein expression, increased CD4^+^ CTLs, and fewer p16^INK4a+^ senescent osteoblasts in high bone density versus low‐density samples. Given the potential role of SIRT1 in regulating immune responses through the deacetylation of key transcription factors and considering the observed immune features, it is plausible that SIRT1‐mediated enhanced immune surveillance in the bone microenvironment contributes to resistance against ageing‐induced OP.

Current research demonstrates that estrogen regulates the expression and function of SIRT1 in osteoblasts through multiple pathways, thereby influencing bone metabolism, delaying cellular senescence, and playing a pivotal role in the prevention and treatment of osteoporosis. For example, in primary osteoblastic cells, exposure to 17β‐estradiol improved cell viability and activated both ERα and SIRT1 expression.^[^ ^36^ ^]^ Further study demonstrated that up‐regulation of SIRT1 induced by 17β‐E2 could promote autophagy via the AMPK‐mTOR pathway and inhibit apoptosis via the FOXO3a activation in osteoblasts.^[^ ^37^ ^]^

Besides, estrogen has been reported to augment the T cell‐dependent immune response, such as CD4^+^ T cell survival and expansion.^[^ ^38^, ^39^ ^]^ However, so far, there have been no reported studies investigating the regulatory effects of estrogen on CD4^+^ CTLs, which warrants in‐depth exploration in future research.

Preclinical studies have indicated a significant role of SIRT1 in immune disease pathogenesis.^[^ ^20^, ^40^ ^]^ Our study revealed that osteoblast‐specific SIRT1‐knockout mice exhibited fewer CD4^+^ CTLs near the bone trabeculae than those of WT mice, implying a strong link between SIRT1 expression in osteoblasts and CD4^+^ CTL distribution in the bone microenvironment. Is it possible that CD4^+^ CTLs contribute to maintaining bone homeostasis and slowing bone ageing by facilitating immune clearance of senescent osteoblasts. A recent study revealed that CD4^+^ CTLs can target the cytomegalovirus glycoprotein (HCMV‐gB) in an MHC‐II‐dependent manner, clearing senescent fibroblasts.^[^ ^10^ ^]^ Interestingly, there is an inverse relationship between CD4^+^ CTL and senescent fibroblast numbers in aged skin. Therefore, we hypothesised that SIRT1 facilitates the “immune clearance” of senescent osteoblasts through CD4^+^ CTLs, thereby maintaining bone homeostasis and decelerating bone ageing. Nonetheless, the detailed mechanism and signalling pathway by which SIRT1 regulates CD4^+^ CTLs requires further investigation.

A large sample analysis found that DAAM2 appears to be a promising target gene for OP therapy.^[^ ^26^ ^]^ However, this study did not investigate the specific upstream and downstream mechanisms of DAAM2. Our study found that SIRT1 inhibits the histone methyltransferase activity of EZH2 by altering its acetylation state, thereby enhancing DAAM2 expression by switching the promoter region of DAAM2 from a repressed to an active state.^[^ ^41^ ^]^ The beneficial effect of DAAM2 on CD4^+^ CTL migration and activation led us to explore its role in OP treatment using an AAV system targeting bone tissue. Our study showed that DAAM2 overexpression ameliorated the progression of OP caused by SIRT1 deficiency in mice by enhancing CD4^+^ CTL activity. in vivo studies revealed that combining CCR5 and CXCR3 antagonists (Maraviroc & AMG 487) inhibited DAAM2's bone mass restoration effect. This suggests that DAAM2 regulates bone homeostasis by increasing the secretion of CCL3, CCL5, and CXCL10, thereby facilitating the migration and activation of CD4^+^ CTLs. Given that DAAM2 functions downstream of Wnt ligands and could activate β‐catenin upstream, β‐catenin activity has been inferred to be linked to the expression of CCL3, CCL5, and CXCL10.^[^ ^25^, ^42^, ^43^, ^44^ ^]^ Therefore, we hypothesised that DAAM2 might regulate the transcription of CCL3, CCL5, and CXCL10 via β‐catenin, which needs further investigation.

In summary, we demonstrated a landscape of the interaction between immune cells and osteoblasts through spatial analysis of the bone microenvironment. Second, we show that, beyond the well‐studied, cell‐autonomous mechanisms of SIRT1, it also exerts a non‐cell‐autonomous function by regulating the osteoblast‐CD4^+^ CTL crosstalk. Finally, we report that DAAM2 is a pivotal downstream effector for SIRT1 to exert immune‐regulatory effects in the bone microenvironment, and that targeting DAAM2 could accurately treat OP by increasing CD4^+^ CTL responses. To the best of our knowledge, this study is the first to identify that DAAM2, a crucial regulator of spinal cord development, maintains the dynamic balance between the production and clearance of senescent osteoblasts in a SIRT1‐regulated manner.

## 4 Experimental Section

### Patient Samples and Processing Methods

Human bone samples were collected from patients who underwent lumbar laminectomy at the Northern Jiangsu People's Hospital of Yangzhou University. Bone samples were obtained from comparable regions of the lumbar spine of 12 patients who underwent lumbar laminectomy with or without a diagnosis of OP (*n* = 6 samples for the osteoporotic group and *n* = 6 samples for the control group). All procedures involving human participants were conducted following the Declaration of Helsinki. The study protocol was approved by the Medical Ethics Committee of Northern Jiangsu People's Hospital (Approval No.: 2023ky232), and written informed consent was obtained from all participants. The samples were trimmed and fixed in 4% PFA for 3 days, and decalcification was implemented using 14% tetra EDTA buffer to pH 7.4 at 4 °C for 1 month. Samples were embedded in paraffin, 4 µm sections were cut onto Leica X‐tra slides, baked at 40 °C for 2 days, and then air‐dried for 2 days.

### Immunostaining and IMC

The sections of bone samples from patients were heated at 68 °C for 1 h and then deparaffinised by incubating them twice in xylene for 10 min each. Sections were rehydrated for 5 min in 95%, 85%, and 75% ethanol at room temperature (RT), followed by heat‐mediated antigen retrieval for 30 min in boiling sodium citrate solution. After naturally cooling to RT, the sections were washed twice with PBS containing 0.5% Tween‐20 and 1% bovine serum albumin (PBS‐Tris buffer (TB)) for 5 min each. The sections were blocked with SuperBlockTM (PBS) Blocking Buffer (37580, Thermo Fisher Scientific) for 30 min at RT. The sections were incubated with the antibody mixture overnight at 4 °C. After these steps, three additional PBS‐TB washes were performed. Ir solution was added dropwise to each tissue to label the cell nuclei and incubated for 30 min at RT, followed by two washes with PBS‐TB and one wash with ddH_2_O. An imaging mass cytometer (Fluidigm, Hyperion) was used to scan the tissue sections and generate multiplexed images. To segment the image data into single‐cell data, the CellProfiler software (Whitehead Institute for Biomedical Research and MIT's CSAIL) was used to obtain mask files, which were fed into the HistoCAT software for UMAP and PhenoGraph analyses and cell neighbourhood analysis. The cellular vicinity of each cell was obtained through windows encompassing the 20 closest neighbouring cells, as gauged by the Euclidean distance between X/Y coordinates. The counts of interactions between the centre and neighbouring cells were characterised for each cell of type A (center) and the average number of neighbours of type B (neighbour) per region of interest within the selected areas. To explore cell‐cell interactions, a permutation test approach for testing interactions implemented in imcRtools (version 1.0.2) was employed to ascertain whether interactions/avoidances between each cell type within each CN occurred more frequently than random occurrences.^[^ ^45^ ^]^

The following primary antibodies were used: anti‐CD3 (1:400, Cell Signalling Technology, #24581SF), anti‐CD4 (1:200, Abcam, #ab181724), anti‐CD8 (1:400, Biolegend, #372902), anti‐CD14 (1:400, Abcam, #ab226121), anti‐CD20 (1:400, Abcam, #ab213033), anti‐CD57 (1:400, BD, #555618), anti‐CD68 (1:800, Biolegend, #916104), anti‐CD163 (1:800, Abcam, #ab215976), anti‐Granzyme B (1:400, Abcam, #ab219803), anti‐ALP (1:100, Abcam, #ab307727), anti‐p16^INK4a^ (1:100, Abcam, #ab186932), and anti‐FoxP3(1:100, Cell Signalling Technology, #74816SF).

### Data Acquisition and Analysis

Data acquisition and preprocessing: In this study, six published single‐cell RNA‐sequencing datasets (GSE212584, GSE171541, GSE200164, GSE120221, GSE157007, and GSE213516) from the NCBI GEO database were downloaded. The sample types included cerebrospinal fluid, spinal cord, lung tissue, and peripheral blood mononuclear cells (PBMCs). Quality control and normalization of the raw count matrices were performed using the Seurat package (version 4.1.0), in R. Low‐quality cells with mitochondrial gene expression greater than 5% and cells with fewer than 200 or more than 5000 detected genes were filtered out. The expression matrices for each sample were log‐normalized, followed by highly variable gene identification and feature selection. Multiple datasets were integrated using the IntegrateData function from the Seurat package to remove batch effects. The “FindIntegrationAnchors” and “IntegrateData” functions were applied to integrate datasets using highly variable genes. The integrated data was used for subsequent analyses.

Cell clustering and annotation: The integrated data underwent PCA dimensionality reduction using the Seurat package, followed by cell clustering using the “FindNeighbors” and “FindClusters” functions (resolution set to 0.5). Cell clusters were manually annotated based on the expression patterns of classic marker genes. CD4^+^ T cells were selected based on high expression of CD4 and CD3E genes, and the CD4^+^ CTL subset was defined by high expression of CD107a (LAMP1).

Correlation analysis of CD4^+^ CTL proportion and Sirtuins expression: The expression abundance of Sirt1‐7 genes (SIRT1, SIRT2, SIRT3, SIRT4, SIRT5, SIRT6, and SIRT7) across tissues and cell types was visualized. The “DotPlot,” “VlnPlot,” and “FeaturePlot” functions from the Seurat package were used to generate gene expression plots for Sirtuins in various tissues and cell types. The proportion of the CD4^+^ CTL subset in each sample was calculated. Spearman correlation analysis was performed to assess the correlation between the proportion of the CD4^+^ CTL subset and the expression levels of Sirt1‐7 genes across tissues. Correlation scatter plots and fitting curves were generated using the ggpubr package, in R. A *p* < 0.05 was considered statistically.

### Animal Studies

SIRT1‐loxP, OC (BGLAP)‐Cre, and WT littermates were obtained from Cyagen Biosciences Inc. (Suzhou, China). SIRT1‐loxP mice were crossed with OC‐Cre mice to generate osteoblast‐specific SIRT1 knockout mice. The C57/BL6 mice used for Ovx modelling and natural ageing were obtained from the Comparative Medical Center of Yangzhou University. Laboratory conditions for mice are as follows: temperature, 22 °C; humidity, 50%; light‐dark cycle, 12 h; water and food freely available. The OP mouse model was established using an 8‐week Ovx protocol. The experimental mice were anaesthetised with isoflurane, and the Ovx mice underwent sterile surgery following a previously described procedure. Both ovaries of ovariectomised mice were removed through midline skin and lateral peritoneal incisions. The skin incision was closed using surgical sutures. The use of animals and experimental protocols was approved by the Animal Care Committee of Yangzhou University (Approval No.: 202311156) following the Institutional Animal Care and Use guidelines.

### Micro‐CT Analysis

After sacrificing the mice, femur bone tissues were collected and fixed in 4% PFA for 24 h. Specimens were scanned using a Bruker Micro‐CT SkyScan 1276 system (Kontich, Belgium). Scan settings are as follows: voxel size 6.534165 µm, medium resolution, 85 kV, 200 µA, 0.25 mm Al filter, and integration time 350 ms. Density measurements were calibrated to the manufacturer's calcium hydroxyapatite (CaHA) phantom. Analyses were performed using the manufacturer's evaluation software. Reconstruction was performed using NRecon (version 1.7.4.2). 3D images were obtained from the contoured 2D images using methods based on the distance transformation of the original grayscale images (CTvox; version 3.3.0). 3D and 2D analyses were performed using the CT Analyser software (version 1.18.8.0). Analyses of bone microarchitecture were performed in an ROI that was 5% of the femoral length from 0.1 mm below the growth plate. The following trabecular bone parameters were quantified: BV/TV, Tb.N, Tb.Th, and Tb.Sp.

### Histology and Immunostaining

Mouse femurs were collected, fixed in 4% PFA for 2 days, and decalcified in 10% EDTA for 14 days. Thereafter, the femurs were embedded in paraffin and serially sectioned longitudinally oriented at a thickness of 4 µm. Paraffin sections were dewaxed twice in dimethylbenzene for 15 min and dehydrated in 100%, 95%, 80%, and 75% ethanol for 5 min each. After 20 min of immersion in 0.5% TritonX‐100, antigen repair was continued in EDTA at 95 °C for another 20 min. After the sections were cooled to room temperature, 3% H_2_O_2_ was added for 15 min to block peroxidase. Goat serum (10%) blocked sections for 1 h. The primary antibody was diluted into an appropriate concentration according to the pre‐experiment results and incubated for 12 h at 4 °C. After washing three times with the primary antibody, horseradish peroxidase‐conjugated secondary antibody (1:200, ABclonal, #AS014) was incubated for 30–60 min at room temperature, according to the manufacturer's instructions. A diaminobenzidine (DAB) kit and haematoxylin were used for immunohistochemical staining. TRAP staining was performed using a commercial kit per the manufacturer's instructions (Sigma, #387A‐1KT) and counterstained with methyl green. IF staining required fluorescent labelling and tyramine signal amplification (AiFang biological, #AFIHC037). The above steps were repeated for antigen repair, and the next round of fluorescent staining was performed. Finally, 4′,6‐diamidino‐2‐phenylindole was used for nuclear IF staining. The following primary antibodies were used: anti‐CD4 (1:100, Cell Signalling Technology, #25229), anti‐perforin (1:100, Cell Signalling Technology, #44865S), anti‐ALP (1:200, Proteintech, #11187‐1‐AP), anti‐ p16^INK4a^ (1:100, Abcam, #ab252788). Images were captured using a confocal microscope (Nikon).

### Small Interfering RNA‐mediated Knockdown of SIRT1 and DAAM2

SIRT1 and DAAM2 knockdown in MC3T3‐E1 cells was achieved by small interfering RNA (siRNA). Initially, three siRNAs targeting different SIRT1 and DAAM2 mRNA regions were tested, and siRNAs with significant knockdown efficiency were detected. The control containing scrambled sequences was transfected into cells using the riboFECTCP Transfection Reagent (RiboBio, #C10511) according to a standard protocol. SIRT1 siRNA and DAAM2 siRNA were purchased from RiboBio Co. Ltd. The effective targeting sequences described above are listed in Table S1 (Supporting Information).

### RNA Isolation and qRT‐PCR

Total RNA was isolated using RNA‐easy Isolation Reagent (Vazyme, #R701). The purity and concentration of the total RNA were assessed using a NanoDrop ND‐1000 spectrophotometre (Thermo Fisher Scientific, USA). For cDNA synthesis, 800 ng of total RNA from each sample was reverse transcribed using a cDNA synthesis kit (Yeasen, #11139ES60). qRT‐PCR was performed with 2×Universal Blue SYBR Green qPCR Master Mix (Servicebio, #G3326) using a Bio‐Rad CFX96 system (Bio‐Rad, USA). mRNA expression was normalised to that of glyceraldehyde‐3‐phosphate dehydrogenase (GAPDH). All experiments were performed with at least three independent biological replicates. Primer sequences are listed in Table S2 (Supporting Information).

### In Vitro Cell Culture and Induction

The MC3T3‐E1 cells were purchased from the cell bank of the Chinese Academy of Science, Shanghai and cultured in the complete medium [α‐minimum essential medium (Gibco, #c12571500BT) containing 10% foetal bovine serum (ExCell Bio, #FND500) and 1% antibiotics (penicillin/streptomycin)] under 5% CO_2_ atmosphere at 37 °C. Primary osteoblasts were isolated from the parietal bones of 7‐day‐old neonatal mice by triple collagenase/II digestion. The cells were cultured in a complete medium containing the components as described previously. For osteoblast differentiation, the complete medium was supplemented with other induction ingredients [ascorbic acid (50 µg mL^−1^) and 5 mm β‐glycerophosphate]. The medium was changed every 3 days, and growth was continued for 21 days. Osteoblasts were fixed in 4% PFA and then stained with 2% Alizarin red S solution (Cyagen Biosciences, #ALIR‐10001) for 30 min at 37 °C. The cells were then imaged under an optical microscope, and the areas of mineralised nodules were calculated using Image‐Pro Plus 6.0 software (Media Cybernetics, USA). All experiments were performed in triplicate to obtain the average data.

### Western Blot Analysis and Immunoprecipitation

Radioimmunoprecipitation assay (RIPA) Lysis Buffer (New Cell & Molecular Biotech, #WB3100) was used to isolate total proteins according to the manufacturer's instructions. Protein concentrations were quantified using a BCA Protein Assay Kit (Beyotime, #P0010). After electrophoresis on SDS‐PAGE, proteins were transferred to polyvinylidene difluoride (Merck Millipore, #IPVH00010) membranes and incubated with the following primary antibodies: anti‐COL1A1 (1:1000, Cell Signaling Technology, #72026S); anti‐RUNX2 (1:1000, Cell Signaling Technology, #12556S), anti‐DAAM2 (1:2000, Abcam, #ab169527), Anti‐SIRT1 (1:1000, Cell Signaling Technology, #8469), anti‐TRAP (1:2000, Abcam, #ab191406), and anti‐CTSK (1:1000, Affinity, #DF6614). Immunoprecipitation is described as the collection of total cell lysates and incubation with anti‐EZH2 antibodies (1:1000, Cell Signaling Technology, #5246) for immunoprecipitation, followed by adsorption onto Protein A/G PLUS‐Agarose (Santa Cruz Biotechnology, #SC‐2003). Immunoprecipitates were separated using SDS‐PAGE and blotted onto polyvinylidene difluoride membranes. The membranes were then incubated with an antibody against acetylated lysine (1:1000; Cell Signalling Technology, #9441). The membranes were then incubated with HRP‐labelled secondary antibodies (1:5000, ABclonal, #AS014, and AS003), and the signals were visualised using Super ECL Detection Reagent (Yeasen, #36208). Finally, band densities were analysed using ImageJ software.

### Lymphocyte Isolation and Flow Cytometry Sorting

After 1‐month‐old mice were sacrificed under isoflurane anaesthesia, they were immersed in 75% ethanol, and the spleens were removed via aseptic surgery. Lymphocytes were separated using Mouse 1×Lymphocyte Separation Medium (DAKEWE, #7211011) according to the manufacturer's instructions. CD3^hi^ CD4^hi^ CD107a^hi^ cells in lymphocytes were considered CD4^+^ CTLs and were sorted using flow cytometry. The brief steps for cell processing before sorting were as follows: first, lymphocytes were resuspended using precooled PBS and then incubated in the dark by adding Fixable Viability Dye eFluor 520 (BD Pharmingen, #564407). This step was terminated after 15 min using a staining buffer, and the flow antibody to be detected was added and incubated in the dark for 20 min. Cells were washed, resuspended in staining buffer, and sorted using a FACS Aria III (BD) instrument. Data were collected and analysed using the BD FACSDiva Software and FlowJo Software. The following antibodies were used: PerCP‐Cy5.5 Hamster Anti‐Mouse CD3e (BD Pharmingen, #551163), APC Rat Anti‐Mouse CD4 (BD Pharmingen, #553051), FITC anti‐mouse CD107a (BD Pharmingen, #558661), and Purified Rat Anti‐Mouse CD16/CD32 (BD Pharmingen, #553141) antibodies.

### Senescence‐Associated β‐Galactosidase (SA‐β‐Gal) Staining

Cells were stained with β‐galactosidase with a Senescence β‐galactosidase Staining Kit (Beyotime, #C0602), according to the manufacturer's protocol. Stained cells were imaged using a Nikon confocal microscope.

### Luminex Liquid Suspension Chip Detection

The concentration of cytokines in cell supernatant was monitored using a Luminex liquid suspension chip, performed by Wayen Biotechnologies (Shanghai, China). A Bio‐Plex Pro Mouse Panel 31‐plex cytokine kit was used according to the manufacturer's instructions. Briefly, the cell supernatant was added to plates embedded with microbeads and incubated for 0.5 h. The corresponding antibodies were added to the wells and incubated for 0.5 h. Streptavidin‐PE was added to each well for 10 min, and the values were read using a Luminex 200 system (Luminex, USA).

### ELISA

Serum and cell supernatant samples were used to measure the absorbance values of substrate chromogenic reactions using ELISA kits (Dreambio, China) in strict accordance with the manufacturer's protocols to quantitatively determine the concentrations of CCL3, CCL5, CXCL10, OCN, P1NP, CTX‐1, and TRAP‐5b.

### RNA Sequencing

Total RNAs of siRNA‐treated MC3T3‐E1 cells were extracted following the manual of TRIzol (Ambion, #15596018). Library preparations were sequenced on an Illumina Novaseq 6000, and 150 bp paired‐end reads were generated (Shanghai Genechem Co.,Ltd). The clean RNA‐seq data were aligned to the reference genome using Hisat2 v2.0.5. featureCounts v1.5.0‐p3 was used to count the reads mapped to each gene. Afterwards, fragments per kilobase of exon per million mapped fragments (FPKM) of each gene were calculated based on the length of the gene and read counts mapped to this gene.

### Luciferase Reporter Assay

According to the UCSC Genome Database (http://genome.ucsc.edu/), multiple peak sequences in the promoter region of DAAM2 in mouse liver tissue can modify H3K27me. Three peak sequences were used as truncated sequences of the promoter DAAM2 (TRUNC0, TRUNC1, and TRUNC2) and transfected into cells using the pGL3‐basic vector. Relative luciferase activity was detected using a luciferase assay kit (YEASEN, #11402) to determine the effects of different truncated sequences and EZH2. The PGL3‐basic plasmid served as the negative control. Data were normalised to *Renilla* luciferase activity.

### Bone‐Targeted Recombinant AAV Vector Injection and Bone‐Targeting Assay

AAV‐DSS vectors carrying DAAM2 (AAV‐DAAM2) were constructed by Genechem Co.,Ltd. (Shanghai, China). For AAV‐DAAM2 packaging, the CAG‐NM_001008231‐3flag‐SV40 PolyA vector was used. Empty AAV vectors encoding eGFP (AAV‐Ctrl) were used as controls. AAV‐DSS vectors were injected into different groups of 8‐week‐old mice through the tail. The titre of these AAV vectors was 2 × 10^10^ vg mL^−1^. All mice were sacrificed 8 weeks after surgery, and bone tissue was collected for further analysis. To test the ability of AAV‐DSS vectors to target bone tissue during systemic delivery in vivo, the tissue distribution of AAV‐DSS was assessed using PerkinElmer optical imaging (PerkinElmer, USA) or by monitoring eGFP expression in frozen sections 2 weeks after AAV‐Ctrl was injected.

### Statistics

Statistical analysis was performed using an unpaired, two‐tailed Student's *t*‐test to compare the two groups. Data were analysed using GraphPad Prism software, and *p* < 0.05 indicated statistical significance.

## Conflict of Interest

The authors declare no conflict of interest.

## Author Contributions

Y.X.W., L.W., and K.Y. designed the research; B.Y., G.F.Z., Y.Z., Y.J., and B.J.R. performed experiments; G.L., D.A.W., J.C., and G.S. analysed and discussed the results; B.Y. wrote the article; J.C.W., W.Y.F., J.H.D, and L.H. provided technical support; X.M.F., J.C., Y.Z.Z., F.H.W. contributed to the discussion of the article; L.W. and H.C. critically edited the manuscript. B.Y. and G.F. Z. contributed equally to this work.

## Supporting information



Supporting Information

## Data Availability

All data needed to evaluate the conclusions in the paper are present in the paper and/or the Supplementary Materials. Additional data related to this paper may be requested from the authors.
