# Supplementary material for: Osteoblast‐CD4+ CTL Crosstalk Mediated by SIRT1/DAAM2 Axis Prevents Age‐Related Bone Loss
Source: Adv Sci (Weinh). 2025 Jul 26;12(39):e01170. doi: 10.1002/advs.202501170 (PMC12533366; doi:10.1002/advs.202501170)

**Supplemental information titles and legends**

**
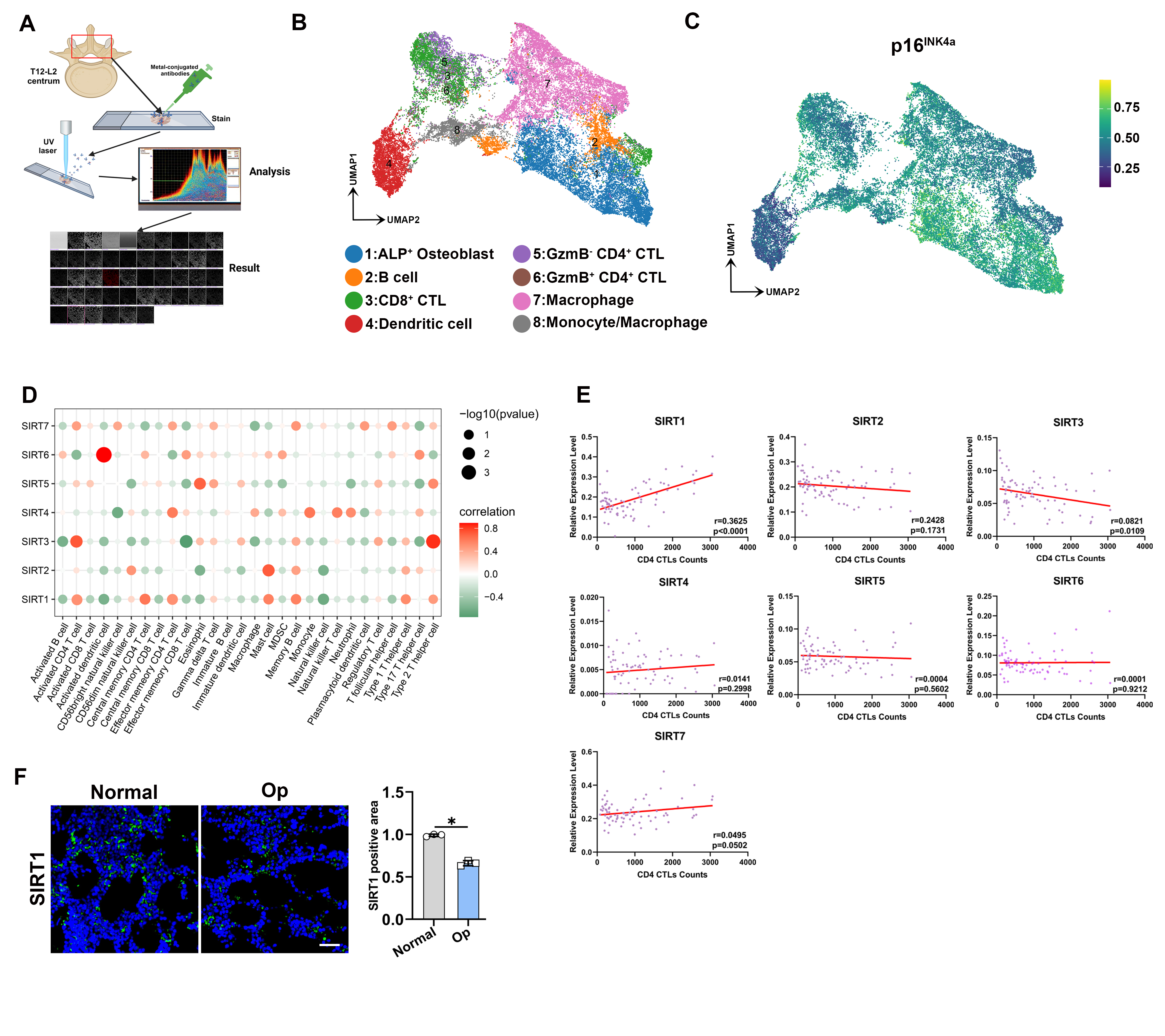
**

**Figure S1. SIRT1 expression may be pivotal in the distribution of CD4^+^ CTLs**

(**A**) Workflow of imaging mass cytometry (IMC). Samples from patients with OP and healthy donors were analysed via IMC. IMC images went through preprocessing before cell segmentation, followed by batch effect removing and downstream bioinformatics analysis. (**B**) UMAP plots were based on the single-cell data extracted from IMC images. 8 clusters of cells from normal and osteoporotic samples were defined according to their markers. (**C**) FeaturePlot of senescence marker p16^INK4a+^ in UMAP plots. (**D**) Correlation between sirtuin mRNA expression levels and immune cell infiltration. (**E**) Correlation of sirtuin family with CD4^+^ CTLs. (**F**) Representative IF staining and quantitative analyses of SIRT1 in human normal and OP bone samples. (scale bar, 50 μm) (Normal, n = 3; Op, n = 3). Data are compared with the control group as the mean ± SD. Statistical significance: **p* < 0.05.

**
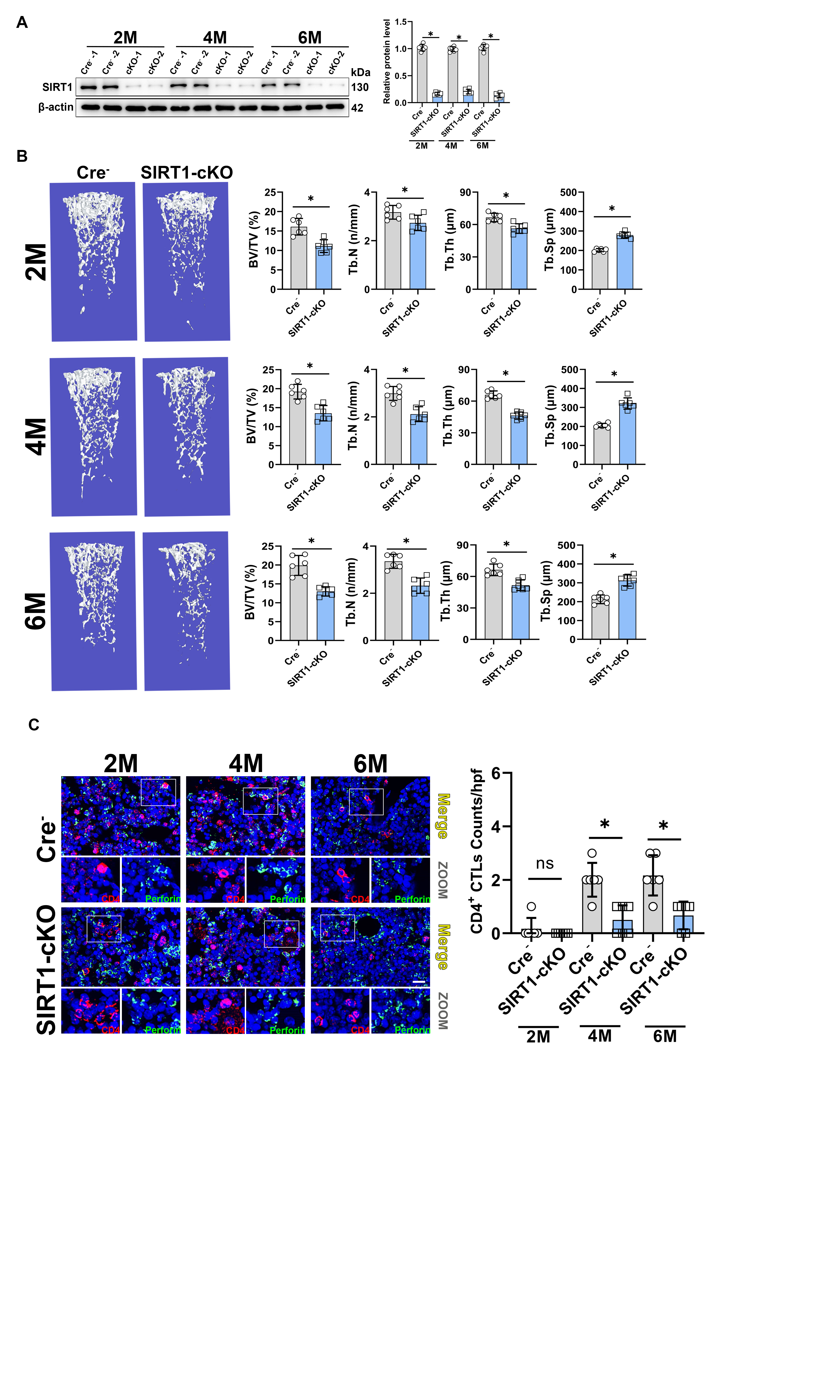
**

**Figure S2. Assessment of bone mass loss and CD4⁺ CTLs distribution in the bone microenvironment of SIRT1-cKO mice at different months**

(**A**) Representative western blots of SIRT1 expression in bone samples of mice and its quantification (n = 6). (**B**) Representative Micro-CT images and quantitative analyses of the distal femora of mice at different months (n = 6). (**C**) Representative images and cell counts of CD4⁺ CTL (scale bar, 50 μm). Data are compared with the control group as the mean ± SD. Statistical significance: **p* < 0.05.

**
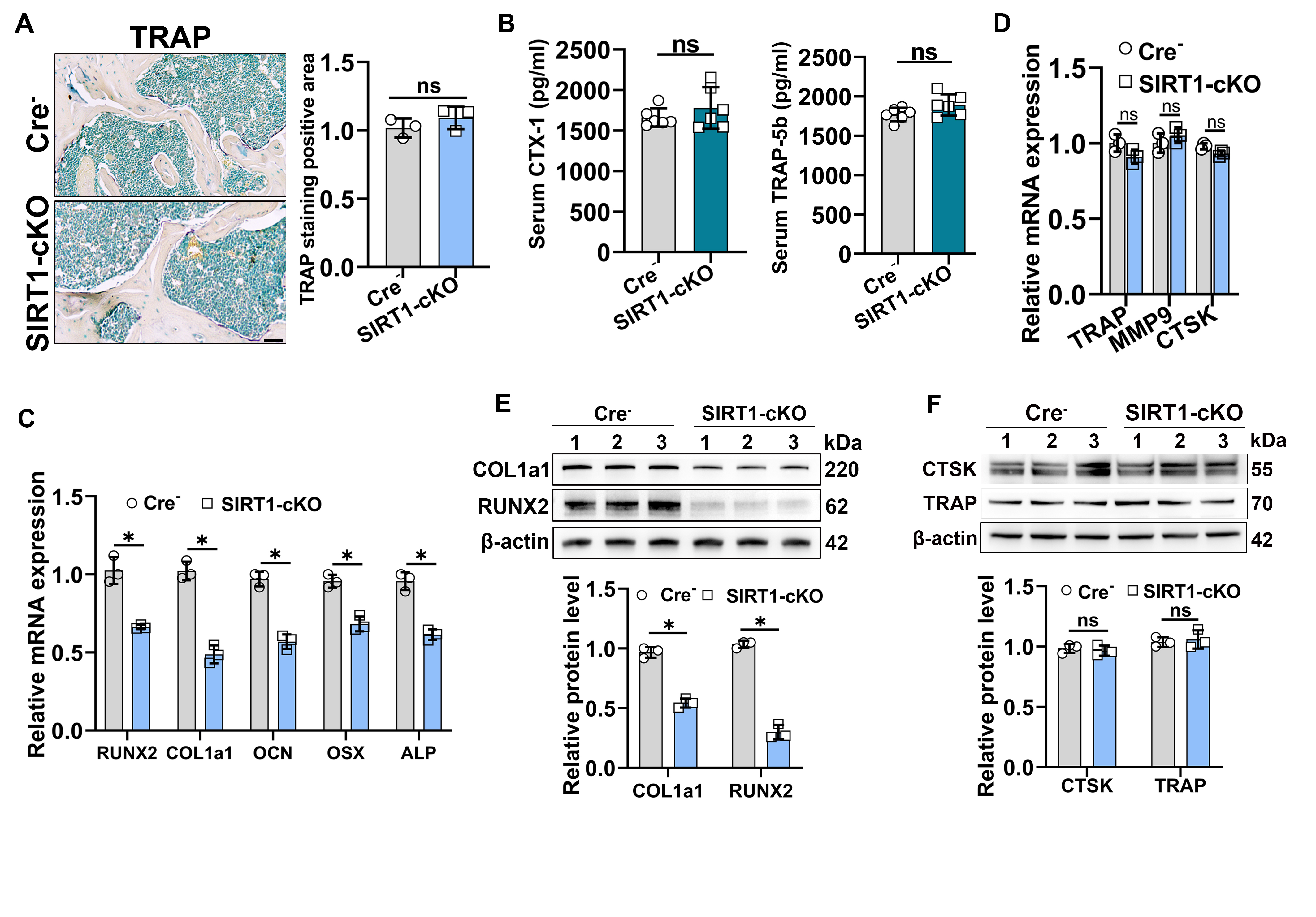
**

**Figure S3. Histological staining and related markers of bone formation and resorption**

(**A**) Representative images of IHC staining for detecting the TRAP expression and quantitative analyses of TRAP staining positive area (scale bar, 50 μm) (n = 3). (**B**) Serum ELISA for bone resorption markers (CTX-1 and TRAP-5b) (n = 6). (**C**) qRT-PCR of the relative osteoblast marker gene (RUNX2, Col1a1, OCN, OSX, and ALP) mRNA levels of the groups indicated (n = 3). **(D)** qRT-PCR of relative osteoclast marker gene (TRAP, MMP9, and CTSK) mRNA levels in the groups (n = 3). (**E**) Representative western blots of Col1a1 and RUNX2 expression in bone samples of mice and its quantification (n = 3). (**F**) Representative western blots and quantification of CTSK and TRAP expression in mouse bone samples (n = 3). Data are compared with the control group as the mean ± SD. Statistical significance: **p* < 0.05.

**
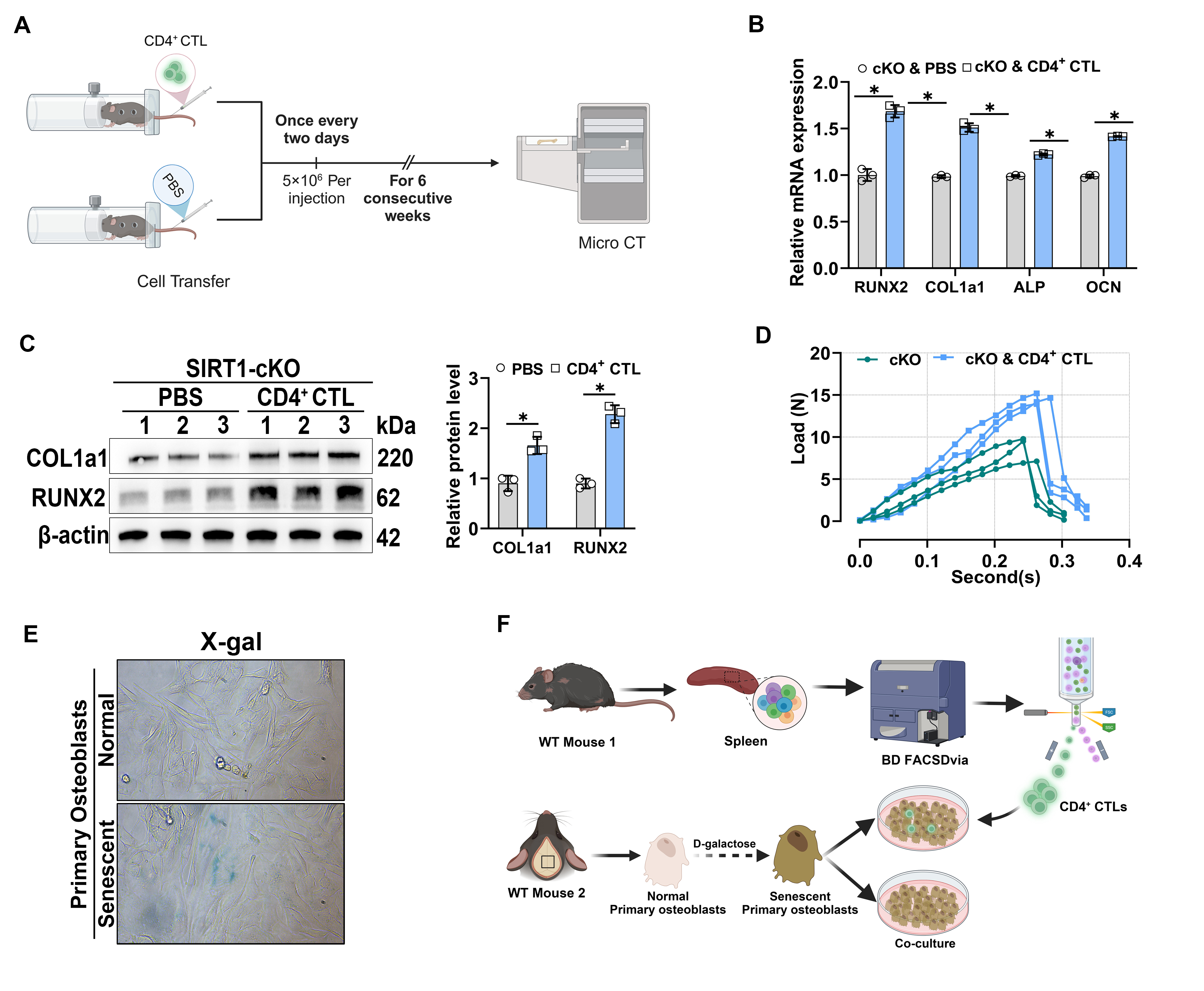
**

**Figure S4. CD4^+^ CTLs were sorted and supplemented to mice and senescent osteoblasts**

(**A**) Schematic representation of SIRT1-cKO mice split into a sham-operated control group and another receiving bidaily tail vein injections of 5×106 CD4^+^ CTLs. (**B**) qRT-PCR of the relative osteoblast marker gene (RUNX2, Col1a1, ALP, and OCN) mRNA levels of the groups indicated (n = 3). (**C**) Representative western blots of COL1a1 and RUNX2 expression in bone samples of mice and its quantification (n = 3). (**D**) Stress test used to detect the femoral cortex ability to withstand stress (n = 3). (**E**) Representative senescence-associated β-galactosidase (SA-β-Gal or X-gal) staining of normal and senescent primary osteoblasts. (**F**) Schematic representation of CD4+ CTL isolated from splenic lymphocytes via flow cytometry and co-cultured with senescent primary osteoblasts. Data are compared with the control group as the mean ± SD. Statistical significance: **p* < 0.05.

**
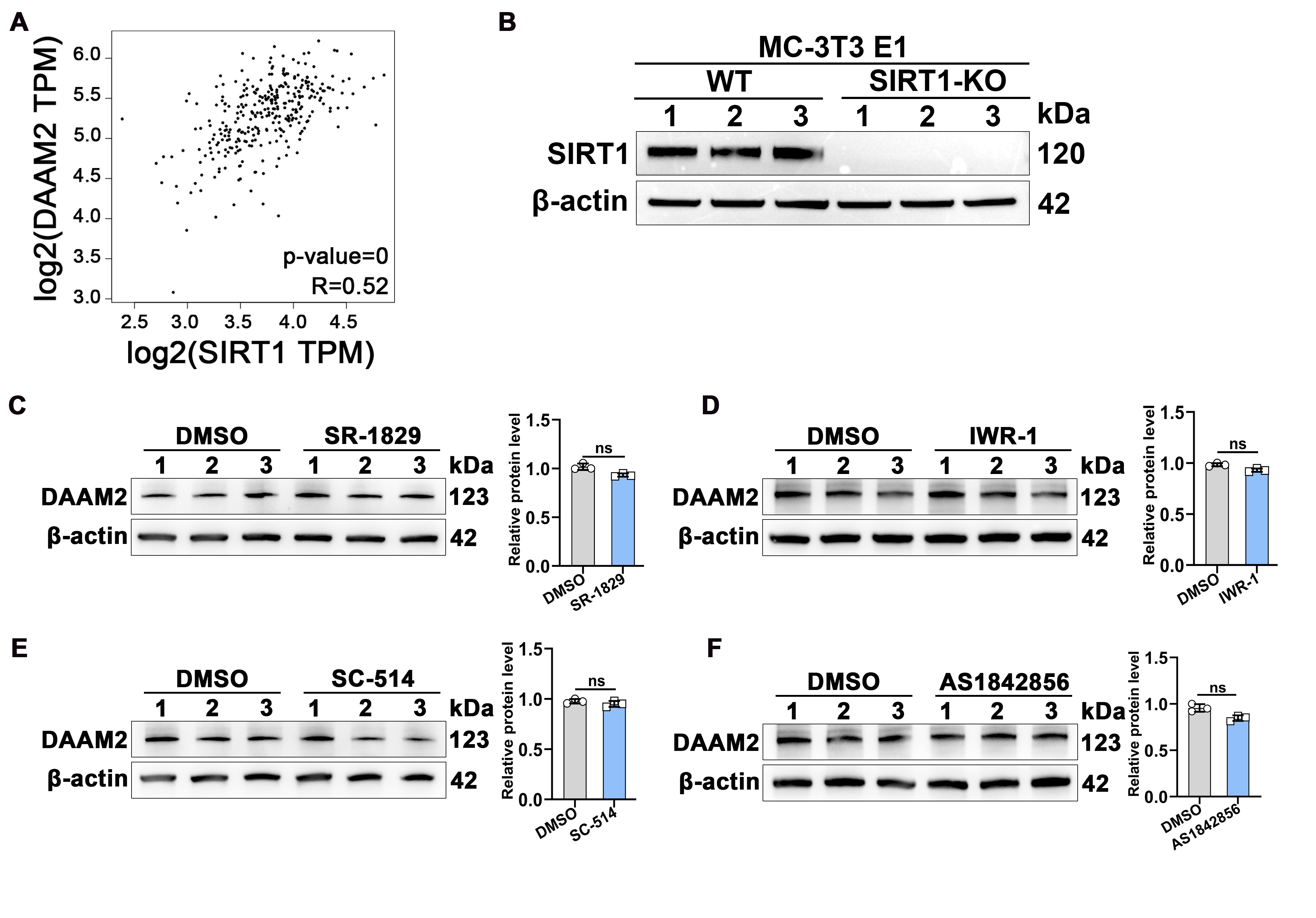
**

**Figure S5. SIRT1 regulates the expression of DAAM2**

(**A**) Gene Expression Profiling Interactive Analysis 2 (GEPIA2) revealed a correlation between SIRT1 and DAAM2. (**B**) Representative western blots of SIRT1 expression in WT and SIRT1-KO MC3T3-E1 cells (n = 3). (**C**)-(**F**) Representative western blots of DAAM2 expression in MC3T3-E1 cells treated with different inhibitors (SR-1829, IWR-1, SC-514, and AS1842856) and their quantification (n = 3). Data are compared with the control group as the mean ± SD. Statistical analysis: **p* < 0.05.

**
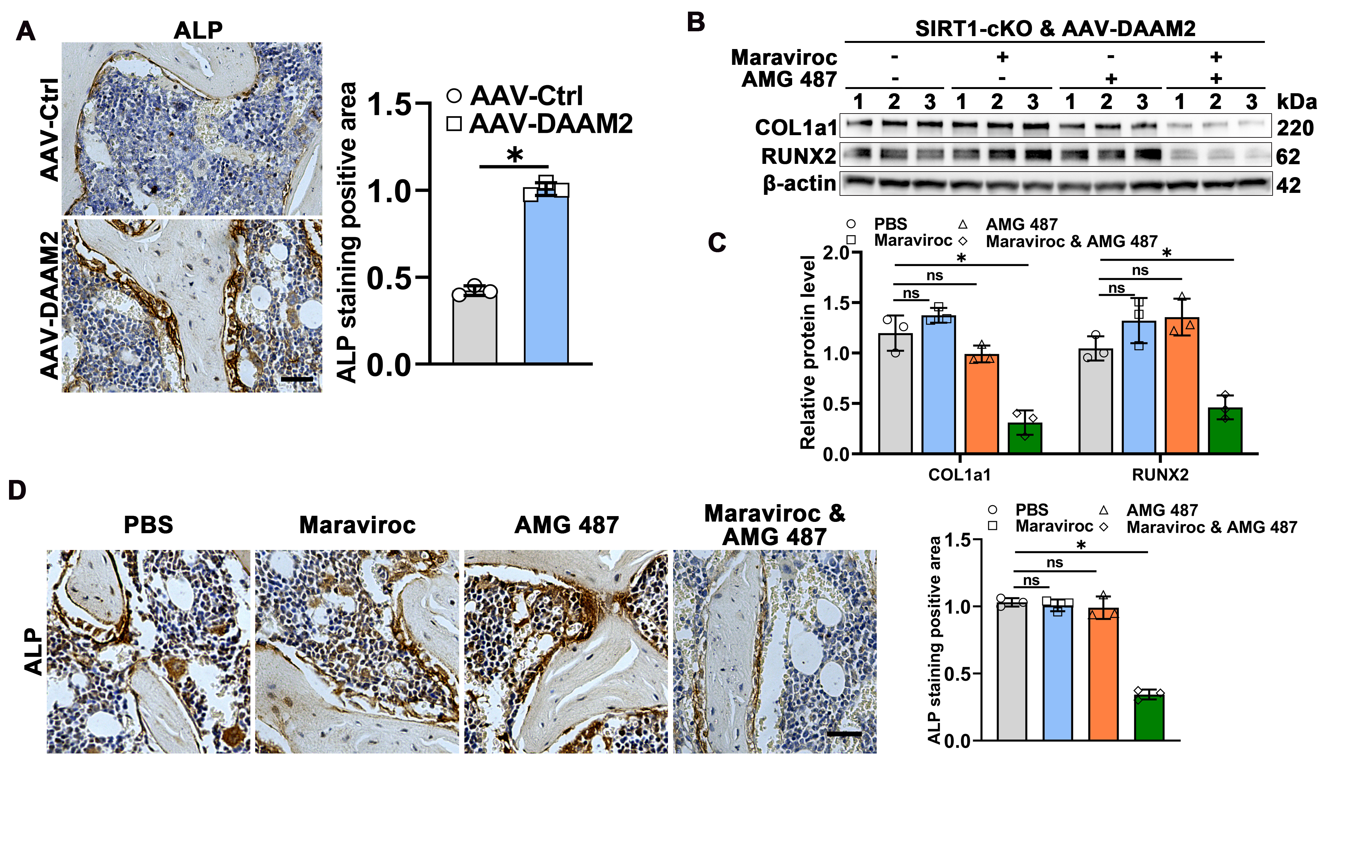
**

**Figure S6. Expression of osteoblasts in the samples detected after different interference measures**

(**A**) Representative images of IHC staining for detecting the ALP expression and quantitative analyses of ALP staining positive area (scale bar, 50 μm) (n = 3). (**B**) and (**C**) Representative western blots of COL1a1 and RUNX2 expression and quantification in bone samples of SIRT1-cKO mice injected with AAV-DAAM2 into four groups: Control, CCR5 antagonist (Maraviroc), CXCR3 antagonist (AMG 487), and mixed antagonists (Maraviroc and AMG 487) (n = 3). (**D**) Representative images of IHC staining for detecting the ALP expression and quantitative analyses of ALP staining positive area (scale bar, 50 μm) (n = 3). Data are compared with the control group as the mean ± SD. Statistical analysis: **p* < 0.05.

**
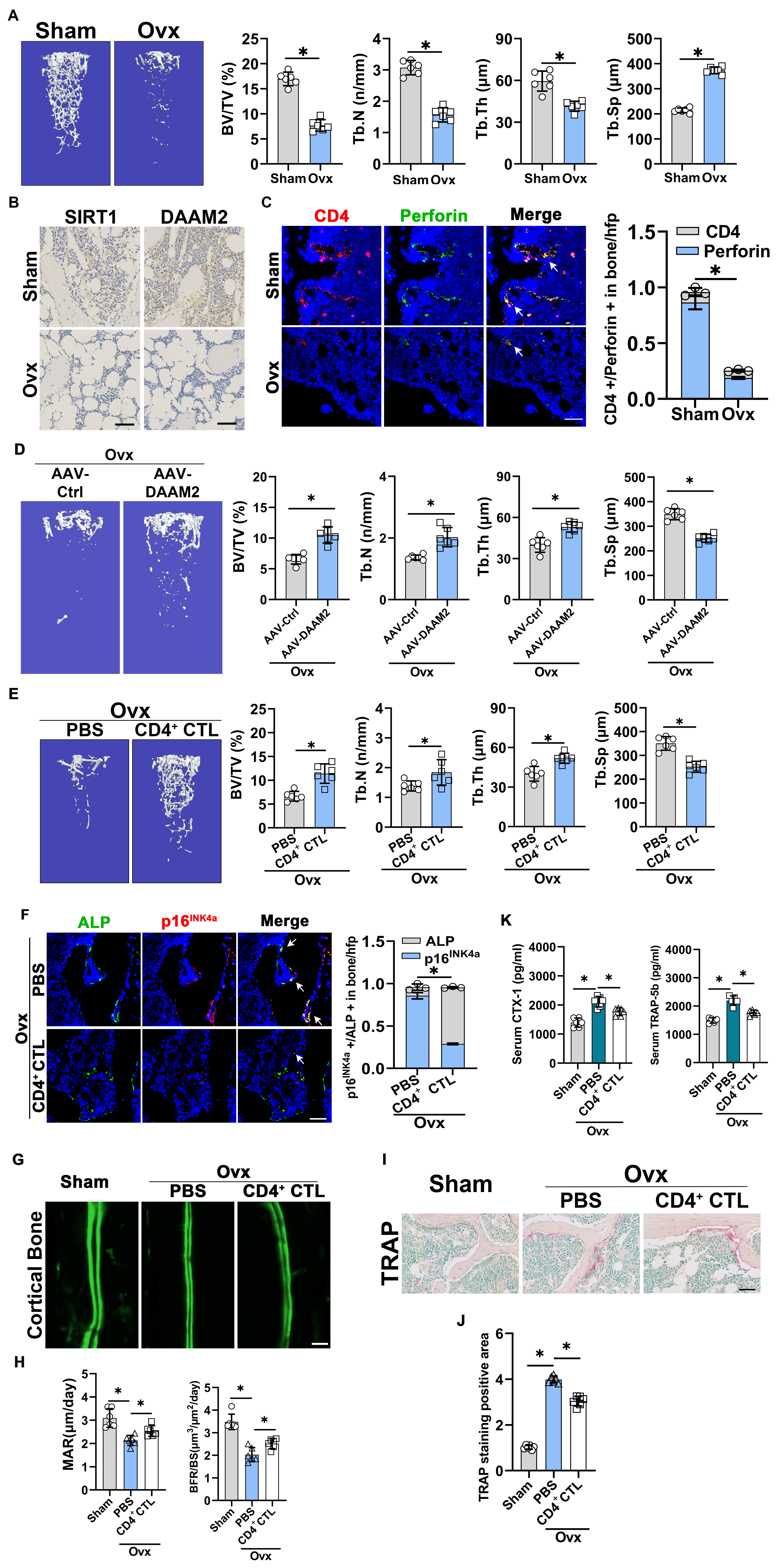
**

**Figure S7. The expression level of SIRT1/DAAM2 and the number of CD4^+^ CTL in the bone microenvironment of Ovx mice were decreased**

(**A**) Representative Micro-CT images and quantitative analyses of the distal femora in Sham and Ovx mice (n = 6). (**B**) Representative images of IHC staining to detect SIRT1 and DAAM2 expression (scale bar, 50 μm) (n = 3). (**C**) Representative IF staining and quantitative analyses of CD4 and Perforin in Sham and Ovx mouse bone samples. (scale bar = 50 μm). (**D**) Representative Micro-CT images and quantitative analyses of the distal femora of Ovx mice injected with AAV-DAAM2 (n = 6). (**E**) Representative Micro-CT images and quantitative analyses of the distal femora of Ovx mice injected with CD4^+^ CTLs (n = 6). (**F**) Representative images and quantitative analyses of the double staining of ALP and p16INK4a in the distal femora of Ovx mice injected with CD4^+^ CTLs (scale bar, 50 μm) (n = 3). (**G**) and (**H**) Dynamic osteogenic index of trabecular bone from the femoral metaphysis in Ovx mice injected with AAV-DAAM2 and corresponding controls, including MAR and BFR determined by calcein double labeling (n = 6). (**I**) and (**J**) Representative images of TRAP staining and quantitative analyses of TRAP staining positive area (scale bar, 50 μm) (n = 6). (**K**) Serum ELISA for bone resorption markers (CTX-1 and TRAP-5b) (n = 6). Data are compared with the control group as the mean ± SD. Statistical significance: **p* < 0.05.

**Table S1. Primer sequences used in siRNA-mediated knockdown of SIRT1 and DAAM2**


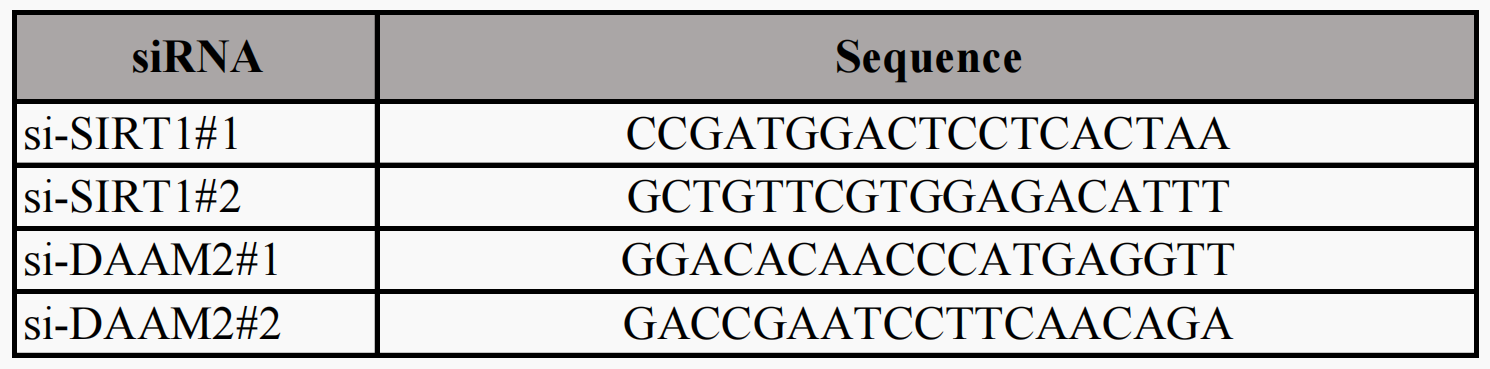


**Table S2. Primer sequences used in qRT-PCR.**


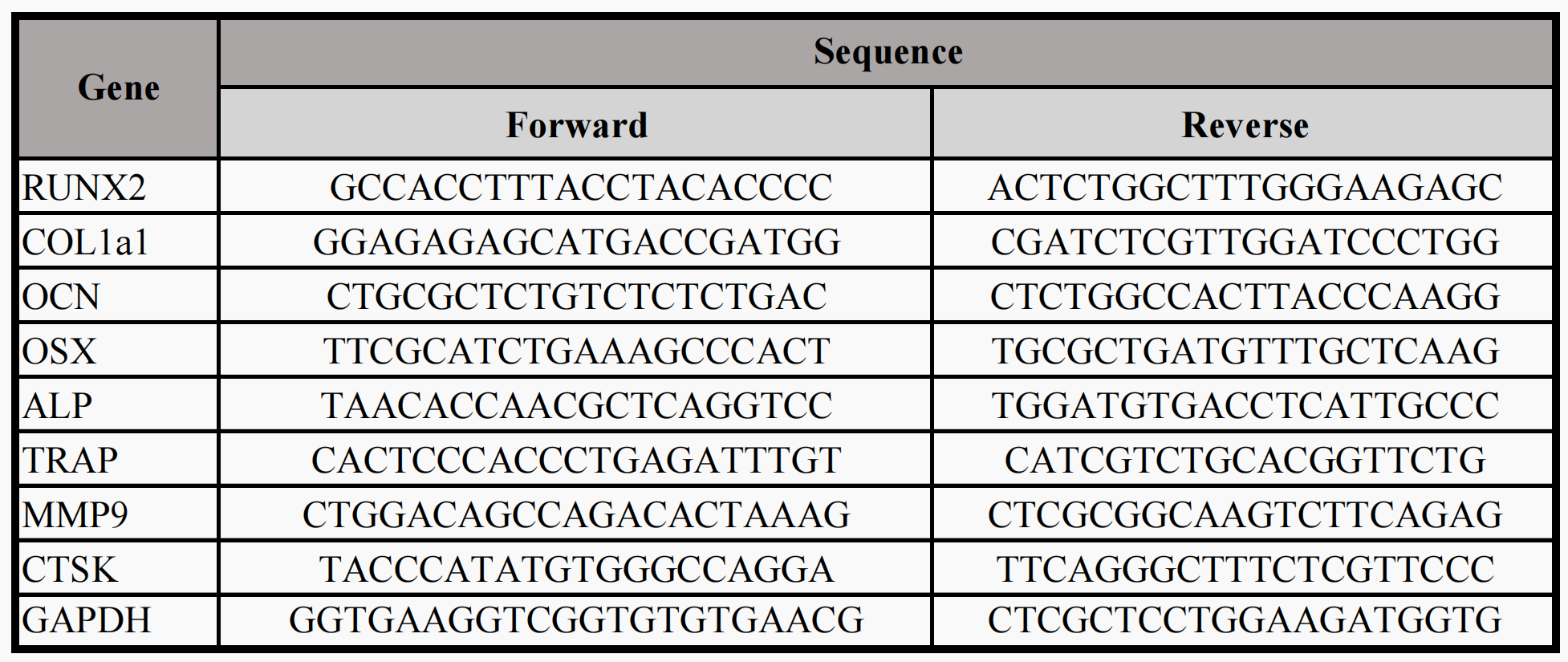

Supplement: Supplementary file 1 — Supporting Information [file ADVS-12-e01170-s001.docx]
